# Temporal trends and future projections of incidence rate and mortality for asthma in China: an analysis of the Global Burden of Disease Study 2021

**DOI:** 10.3389/fmed.2025.1529636

**Published:** 2025-01-29

**Authors:** Xi Chen, Dandan Ma, Hangyu Li, Yilin Liu, Guixing Xu, Xinyu Deng, Qi Li, Junqi Li, Hui Pan

**Affiliations:** ^1^Department of Rehabilitation, West China School of Public Health and West China Fourth Hospital, Sichuan University, Chengdu, China; ^2^Acupuncture and Tuina School, Chengdu University of Traditional Chinese Medicine, Chengdu, China; ^3^Hospital of Chengdu University of Traditional Chinese Medicine, Chengdu, China

**Keywords:** disease burden, asthma, prediction, age-standardized rate, incidence, mortality

## Abstract

**Background:**

Asthma poses a significant public health burden in China, affecting millions with substantial incidence and mortality. Understanding the trends and future projections of asthma incidence and mortality is crucial for healthcare planning.

**Methods:**

We analyzed asthma incidence and mortality data sourced from the Global Burden of Disease (GBD) 2021 study from 1990 to 2021, calculated the age-standardized incidence and mortality rates (ASIR and ASMR) and the estimated annual percentage change (EAPC), meanwhile, employed Joinpoint regression model to assess the trends. The age-period-cohort model was applied to estimate the effects of the age, period, and cohort on the incidence and mortality. Finally, future asthma trends for the next 25 years were predicted utilizing the Bayesian age-period-cohort (BAPC) model.

**Results:**

Over the past three decades, the incidence rate declined in waves while the mortality declined steadily. The ASIR of asthma decreased from 524.81 to 364.17 and the ASMR declined from 5.82 to 1.47. ASIR and ASMR are consistently higher in males than females during this period. The effect attributable to age on incidence was higher for the younger age group while the mortality was higher for older. The period ratio rate of incidence and mortality declined with the calendar year, and the corrections between birth cohort and the risk of incidence and mortality were negative. Our projections indicate that the ASIR and ASMR will continue to decrease by 2046, with expected rates of 330 and 0.69, respectively. Instead, the absolute number of asthma incidence cases and deaths may increase to approximately 4.5 million and 80,000, respectively.

**Conclusion:**

Although asthma incidence rates and mortality have generally declined in China, the burden remains significant, especially among vulnerable groups, with higher rates in males. Continuous monitoring and age-targeted interventions are essential. Future healthcare strategies must address the aging population to manage the projected increase in asthma cases and deaths.

## 1 Introduction

Asthma is a common condition characterized by chronic inflammation of the lower respiratory tract, leading to airflow limitation, airway hyperresponsiveness, and structural changes (1, 2). It poses a significant global public health challenge, with a rising disease burden across many regions. Asthma is associated with the development of chronic obstructive pulmonary disease (COPD) and cardiovascular disease, profoundly impacting patients’ quality of life (3, 4). Although age-standardized disease burden rates have declined over the past 30 years, the incidence, prevalence, and mortality rates continued to rise, reflecting an ongoing severe burden according to Global Burden of Disease (GBD) 2019 (5). The prevalence of asthma in China has increased from 0.69% in 1984 to 5.30% in 2021, with a concerning acceleration in incidence despite lower overall prevalence compared to Western countries (6). But asthma was largely underdiagnosed and undertreated in China, the proportion of asthma patients who used inhaled corticosteroids therapy was only 5.6 to 10.2%, significantly lower than reported rates in some European countries (7). Chronic respiratory diseases remain a leading cause of death and disability worldwide (8). Although asthma-related mortality is lower than that of COPD or lung cancer, severe asthma exacerbations have a significant impact. Vulnerable populations, including the older adult, children, and those with comorbid conditions, face higher risk of fatal outcomes (9, 10). Additionally, asthma is associated with increased psychological stress, with studies showing higher suicide rates among young asthma patients (11).

To assess asthma trends in China, it is essential to consider the nation’s demographic shifts particularly rapid population aging driven by declining fertility and rising life expectancy. China’s fertility rate has declined to notably low levels (12). Meanwhile, the proportion of individuals aged 65 and older rose from 7% in 2000 to 14.2% in 2021, officially classifying China as an aged society (13). These demographic shifts carry notable implications for chronic respiratory diseases, including asthma.

This study aims to analyze trends in asthma incidence and mortality in China from 1990 to 2021 using the latest GBD data and project the asthma burden for the next 25 years. Additionally, we will assess the impact of demographic shifts, especially aging, on asthma trends, providing evidence to inform public health policies and interventions.

## 2 Materials and methods

### 2.1 Study design and data sources

The GBD study offers a tool for quantifying health loss from numerous diseases, injuries, and risk factors (14, 15). Asthma incidence and mortality data in China (without Taiwan province) for this study were retrieved from the Global Health Data Exchange (GHDx).^1^ In GBD 2021, asthma is a chronic lung disease marked by spasms in the bronchi usually resulting from an allergic reaction or hypersensitivity and causing difficulty in breathing. The group defined asthma as a doctor’s diagnosis and wheezing in the past year. The relevant International Classification of Disease and Injuries (ICD-10) codes are J45 and J46. ICD-9 code is 493. Alternative case definitions include self-reported asthma in the past year, self-reported asthma ever, only a doctor’s diagnosis in the past year, and only wheezing in the past year. Vital registration and surveillance data from the cause of death (COD) database were used to estimate asthma mortality.

### 2.2 Statistical analysis

To assess asthma burden trends in China from 1990 to 2021, age-standardized incidence rate (ASIR) and age-standardized mortality rate (ASMR) were calculated. The estimated annual percentage change (EAPC) was used to evaluate age distribution differences across populations. To account for heterogeneity due to sampling error and non-sampling variance, an uncertainty interval (UI) analysis was conducted. The 95% UI was derived from 1,000 samples of the posterior distribution at each modeling step, with the 2.5th and 97.5th percentiles reported for each estimate.

Joinpoint regression model, conducted by Joinpoint software (version 5.2.0), was employed to analyze time trends systematically from 1990 to 2021 and assess the statistical significance of trends between join-points. To quantify the direction and magnitude of these trends, the annual percent change (APC) and average annual percentage change (AAPC) were calculated with confidence interval (CI). The APC evaluates changes within specific periods, while AAPC provides an overall measure of overall changes over time (16). Statistical significance was set at a two-tailed *p*-value <0.05.

The age-period-cohort model is a sophisticated research method that enhances traditional analyses in health and socio-economic studies, particularly for examining trends in chronic disease incidence and mortality. It captures disease trends by considering age, period, and cohort effects. The National Cancer Institute’s Age-Period-Cohort web tool was used for model fitting (17). In this framework, the age and period intervals must be consistent, thus, the analysis was conducted using data from 1992 to 2021, divided into six continuous 5-year periods (1992–1996 to 2017–2021). Age groups were also defined in 5-year intervals, ranging from under-5-year age to over-95-year age.

We predicted asthma-related new cases and deaths from 2022 to 2046 using the Bayesian age-period-cohort (BAPC) model with integrated nested Laplace approximations (INLA), based on asthma burden data from the GBD database from 1990 to 2021. The analysis was performed in R (version 4.4.1) using the “BAPC” (version 0.0.36) and “INLA” (version 24.06.27) packages. Data visualization was carried out with the “ggplot2” (version 3.5.1) package.

## 3 Results

### 3.1 Description of asthma incidence and mortality in China

Between 1990 and 2021, China experienced a significant decline in both all-age asthma incidence and mortality (Table 1). The number of new asthma cases per 100,000 population decreased from 459.22 (95% UI, 394.46–637.92) to 276.57 (95% UI, 223.74–361.00), reflecting a 39.77% reduction. Similarly, deaths owing to asthma declined from 3.08 (95%UI, 2.43–4.42) to 1.84 (95%UI, 1.45–2.24), representing a 40.26% decrease. Corresponding trends were observed in the ASIR, which decreased from 524.81 (95% UI, 421.31–672.76) to 364.17 (95% UI, 283.22–494.10), and in the ASMR, which dropped from 5.82 (95% UI, 4.46–8.50) to 1.47 (95% UI, 1.15–1.79).

**Table 1:** Incidence rate and mortality of asthma in China from 1990 to 2021.

Disease burden, per 100,000 No, (95%UI)	1990	2021
All-age incidence case		
Both	495.22 (394.46,637.92)	276.57 (223.74,361.00)
Male	530.89 (421.17,690.47)	312.56 (251.83,411.59)
Female	457.23 (367.07,578.61)	238.84 (195.13,306.20)
ASIR		
Both	524.81 (421.31, 672.76)	364.17 (283.22, 494.10)
Male	561.78 (448.43, 725.78)	404.54 (312.57, 547.88)
Female	485.03 (392.61, 617.31)	319.56 (248.24, 429.99)
EAPC of incidence cases, 1990–2021		
Both	−1.17(−1.56, −0.78)	
Male	−1.01(−1.37, −0.64)	
Female	−1.40(−1.83, −0.97)	
Death cases		
Both	3.08 (2.43, 4.42)	1.84 (1.45, 2.24)
Male	3.22 (2.30, 5.24)	2.03 (1.42, 2.65)
Female	2.94 (1.84, 4.12)	1.65 (1.13, 2.31)
ASMR		
Both	5.82 (4.46, 8.50)	1.47 (1.15, 1.79)
Male	7.11 (4.95, 12.28)	1.95 (1.38, 2.50)
Female	4.96 (3.10, 7.21)	1.14 (0.78, 1.59)
EAPC of death cases, 1990–2021		
Both	−4.69(−4.93, −4.45)	
Male	−4.21(−4.44, −3.97)	
Female	−5.22(−5.51, −4.92)	

Both males and females exhibited similar trends in asthma incidence and mortality over the past decades, with higher rates observed in males. In males, new asthma cases decreased from 530.89 (95%UI, 421.17–690.47) in 1990 to 312.56 (95%UI, 251.83–411.59) in 2021, while the deaths decreased from 3.22 (95%UI, 2.30–5.24) to 2.03 (95% UI, 1.42–2.65). For females, new cases declined from 457.23 (95% UI, 367.07–578.61) to 238.84 (95% UI, 195.13–306.20), and mortality dropped from 2.94 (95% UI, 1.84–4.12) to 1.65 (95% UI, 1.13–2.31). Regarding age-standardized rate, both ASIR and ASMR were consistently higher in men than in women throughout the study period. Specifically, the ASIR for men decreased from 561.78 (95% UI, 448.43–725.78) to 404.54 (95% UI, 312.57–547.88), while for women, it decreased from 485.03 (95% UI, 392.61–617.31) to 319.56 (95% UI, 248.24–429.99). Both genders experienced a decline in ASMR, with a notable reduction in men from 7.11 (95% UI, 4.95–12.28) to 1.95 (95% UI, 1.38–2.50), and in women from 4.96 (95% UI, 3.10–7.21) to 1.14 (95% UI, 0.78–1.59).

From 1990 to 2021, the EAPC for asthma incidence in the entire population was −1.17 (95%UI, −1.56 to −0.78). Regard to gender, a greater annual reduction in incidence was observed among females. The EAPC for asthma mortality was −4.69 (95%UI, −4.93 to −4.45) for the overall population, and a more significant annual decrease in mortality was also observed in females than in males.

**Figure 1:**
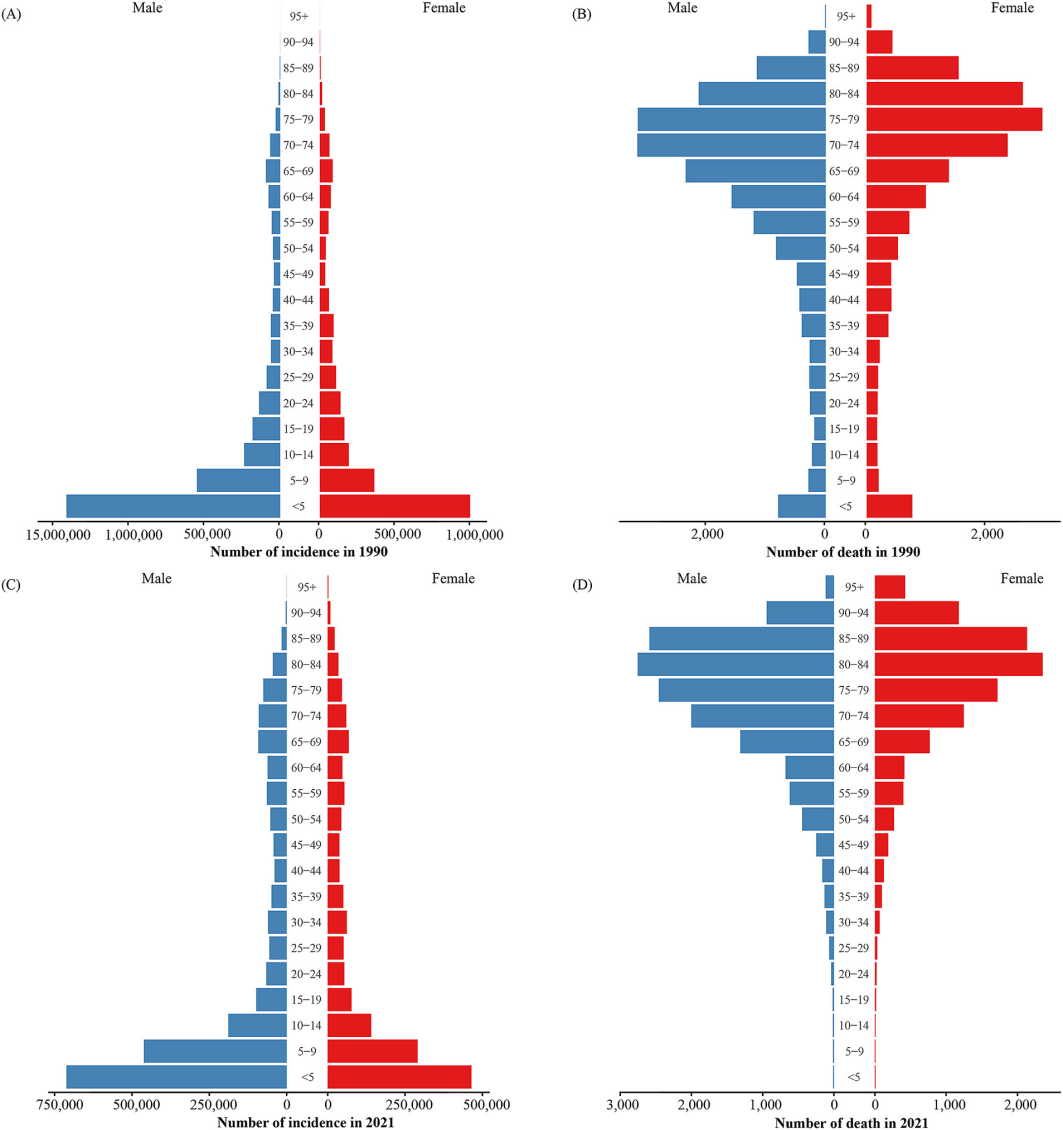
Number of asthma incidence and mortality by age and sex groups in 1990 and 2021. **(A)** Number of incidence cases in 1990. **(B)** Number of death cases in 1990. **(C)** Number of incidence cases in 2021. **(D)** Number of death cases in 2021.

**Figure 2:**
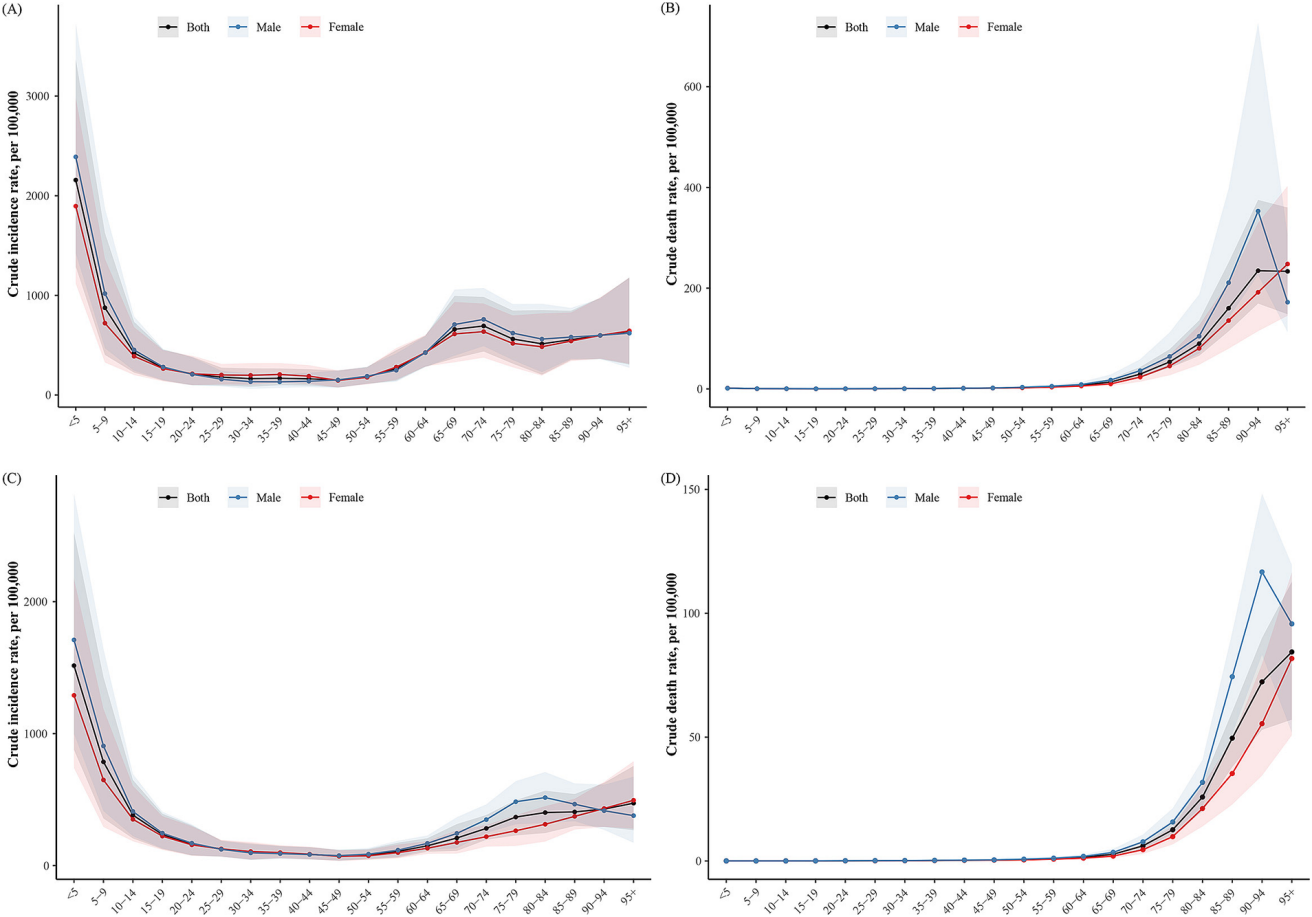
The crude rates of incidence and mortality for asthma in China in 1990 and 2021. **(A)** Crude incidence rate of asthma in 1990. **(B)** Crude death rate of asthma in 1990. **(C)** Crude incidence rate of asthma in 2021. **(D)** Crude death rate of asthma in 2021.

Asthma incidence and mortality varied across age groups. In both 1990 and 2021, the highest incidence occurred in the under-5 age group, with over 50% of cases in 1990 occurring in children under 10 years, and the majority in 2021 occurring in those under 15 years, highlighting the disease’s predominance in children and adolescents. Incidence also increased in individuals over 60 years of age (Figures 1A,C), as reflected in the crude incidence rates (Figures 2A,C). The age group with the highest number of deaths shifted from 75–79 years in 1990 to 80–84 years in 2021. In 1990, 72.28% of deaths were in individuals aged 60 or older, rising to 88.04% by 2021. Deaths in children under 5 decreased from 4.32% in 1990 to less than 0.1% in 2021 (Figures 1B,D). Mortality rates were significantly higher in older age groups, with a notable drop in death rates for males over 95 years, observed in both 1990 and 2021 (Figures 2B,D).

### 3.2 Temporal trends in incidence and mortality in China

**Figure 3:**
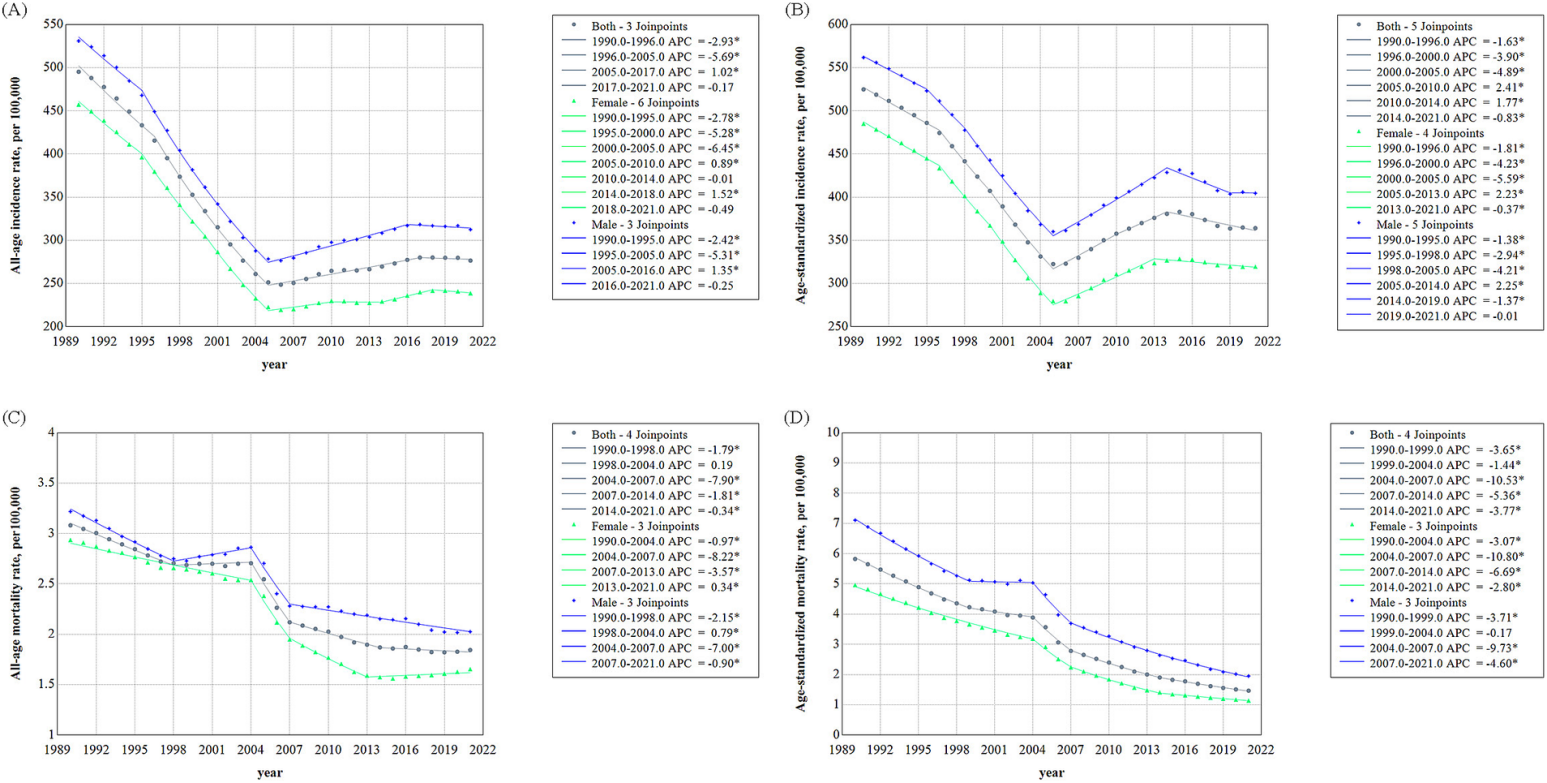
Trends of asthma incidence and mortality in China, 1990–2021. **(A)** Asthma incidence rate. **(B)** Age-standardized asthma incidence. **(C)** Asthma mortality rate. **(D)** Age-standardized asthma mortality.

To investigate changes in asthma incidence and mortality in China from 1990 to 2021, a Joinpoint regression analysis was performed (Figure 3). Overall, both incidence and mortality exhibited a downward trend during this period.

Prior to 2005, the crude incidence rate decreased significantly, with the most notable decrease between 1996 and 2005. After 2005, the downward trend ceased, with a rise in incidence at an APC of 1.02%. However, the decline resumed after 2017. The AAPC for incidence over the entire period was −1.89%. Both male and female incidence rates followed similar trends, but a significance test revealed more join-points and greater fluctuations in females, particularly after 2005. Notably, after 2005, the crude incidence rate increased more in males than females (Figure 3A). Similarly, the ASIR for the total population also declined significantly until 2005, then began to rise. From 2010, the decline resumed, continuing through 2021. The AAPC for incidence over the entire period was −1.21%. Both male and female ASIRs followed the same general trend, with the gender gap in ASIR remaining relatively stable (Figure 3B).

The crude mortality rate of the total population exhibited a consistent downward trend, with an AAPC of −1.70%. A slight increase was observed between 1998 and 2004, though not statistically significant. The most substantial decline occurred between 2004 and 2007, with an APC of −7.90%. Analysis by gender revealed distinct trends. Female mortality declined continuously before 2004, while male mortality reached its lowest point in 1998, followed by a slight increase. Between 2004 and 2007, both male and female mortality rates declined sharply. However, after 2007, the rate of decline slowed, and female mortality showed a slight increase in 2013 through to 2021 (Figure 3C). The ASMR steadily decline from 1990 to 2021 for both the overall population and by gender. The AAPC for the entire population was −4.40%, indicating a significant reduction. While fluctuations occurred, the general trend was a decline, with a modest decrease of −1.44% between 1999 and 2004. After 2004, the decline accelerated, reaching an APC of more than −10%, before smoothing out in subsequent years. The most significant change in the ASMR for males occurred in 2004, following a slight decrease before that. For females, the steepest decline occurred in the decade after 2004. Unlike the ASIR, the gap between male and female ASMR narrowed, particularly after 2007 (Figure 3D).

### 3.3 Age-period-cohort model analysis of asthma incidence and mortality in China

**Figure 4:**
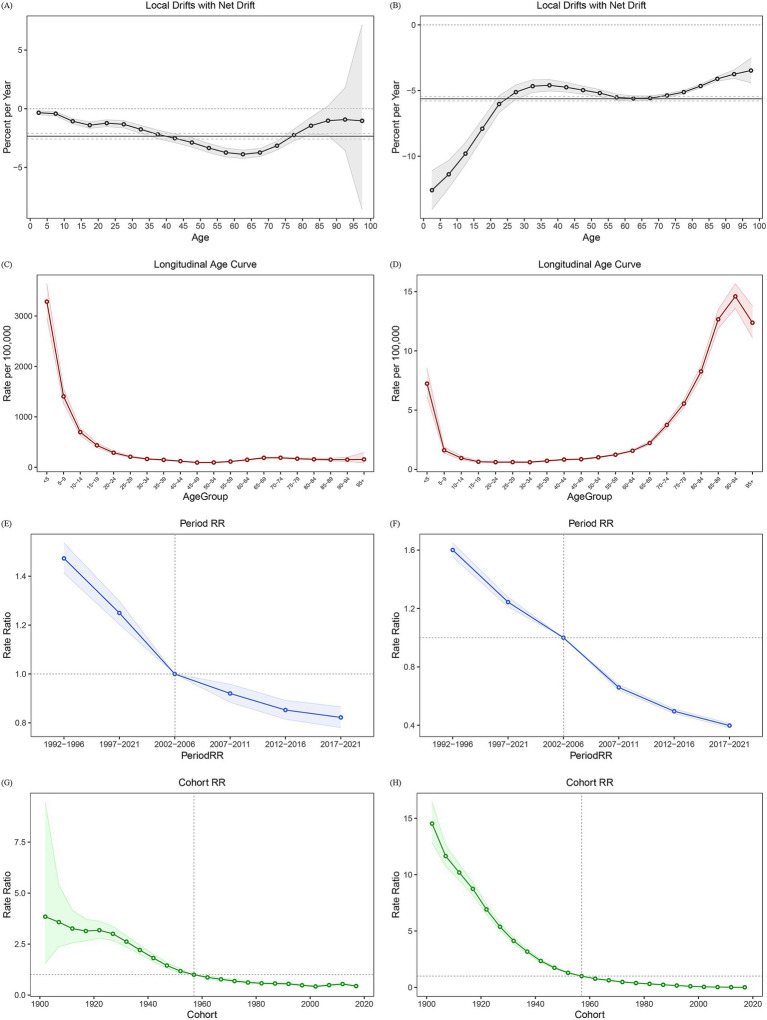
Age-period-cohort effects of asthma incidence and mortality in China, 1990–2021. **(A)** Local drifts and net drift of incidence. **(B)** Local drifts and net drift of mortality. **(C)** Longitudinal age curve of incidence. **(D)** Longitudinal age curve of mortality. **(E)** Period RR curve of incidence; **(F)** Period RR curve of mortality. **(G)** Cohort RR curve of incidence. **(H)** Cohort RR curve of mortality. RR: ratio rate.

An age-period-cohort model analysis was conducted to understand the independent effects of age, period, and birth cohort on asthma incidence and mortality. Local drift and net drift were calculated to illustrate the linear trends after adjusting for period and cohort effects. Net drift reflected the overall trend across all age groups, while local drift represented the trends within specific age groups. Between 1990 and 2021, the net drift for asthma incidence in China was −2.34% per year, and for mortality, it was −5.63% per year. The local drift for incidence initially decreased, then increased in older age groups, but remained negative throughout (Figure 4A). The greatest decline occurred in the under-5 age group, followed by a gradual decrease until the 20–25 age group, where a slight increase was observed. For those over 25 years, local drift continued to decline, reaching its lowest point in the 60–65 age group, before rising again in the 90–95 age group. In contrast, the local drift for mortality showed a different pattern but remained negative (Figure 4B). The lowest local drift for mortality was observed in the under-5 age group, increasing to the 35–40 age group, and then declining in the 60–65 age group. Mortality drift increased again for those aged 80 and above. A similar trend was observed in both male and female local drifts for incidence and mortality, with more significant changes seen in females, particularly for incidence (Supplementary Figures S1, S2).

After adjusting for period and cohort effects, we found that the age effect had a greater impact on asthma incidence in younger age groups (Figure 4C). While mortality was more strongly influenced by age in older groups, except for those under 5 years (Figure 4D). Gender-specific analysis showed a similar trend for incidence. However, a sharp decline in age attribution to mortality was observed in males over 95 years, with values halving compared to the previous age group. In females, age attribution to mortality was nearly six times higher in the under-5 group compared to older age groups, resulting in a notable shift in the longitudinal age curve for both the under-5 and over-95 age groups.

After controlling for age and cohort effects, the period effects on incidence and mortality were assessed using the ratio rates, with the median level for the period 2002–2006 as the baseline. The period effect of incidence declined from 1.47 in 1992–1996 to 0.82 in 2017–2021 (Figure 4E), and mortality risk dropped from 1.60 in 1992–1996 to 0.40 in 2017–2021 (Figure 4F). While the trends in period effects on incidence and mortality were consistent across gender, the decline in these ratios was more pronounced in females than in males.

After controlling for age and period effects, cohort effects on asthma incidence and mortality were assessed using the median level of the 1957–1961 cohort as the baseline. Cohort risk for incidence was significantly elevated in earlier birth cohorts and declined in more recent cohorts (Figure 4G), a pattern also observed for mortality (Figure 4H). Gender-specific analysis revealed higher incidence and mortality rate ratios in early birth cohorts for females compared to males within the same cohorts.

### 3.4 Forecasting the incidence and mortality of asthma in China from 2022 to 2046

**Figure 5:**
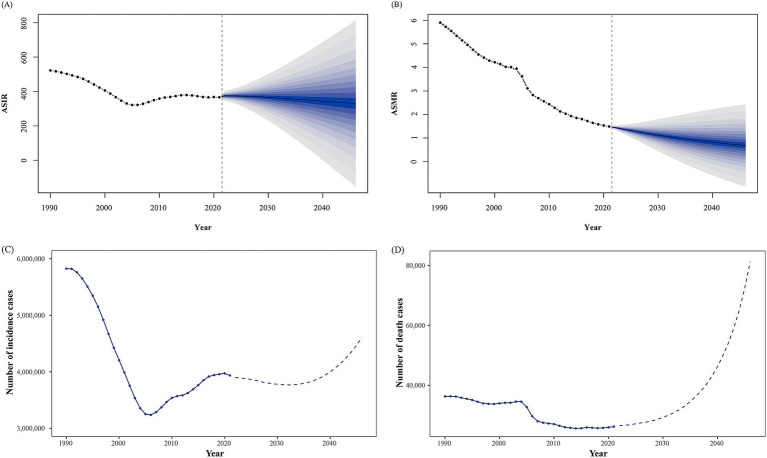
Trends in asthma incidence and mortality observed and predicted from 1990 to 2046. **(A)** Trends of age-standardized incidence rate (ASIR). **(B)** Trends of age-standardized mortality rate (ASMR). **(C)** Trends for the number of incidence cases. **(D)** Trends for the number of death cases.

The BAPC model was employed to project trends in asthma incidence and mortality over the following 25 years. According to the model, asthma incidence is expected to decline continuously, reaching approximately 330 cases per 100,000 people by 2046 (Figure 5A; Supplementary Table S5). New cases are projected to hit a low point in 2032 before gradually increasing, with the total number of cases rising to around 4.5 million by 2046 (Figure 5C; Supplementary Table S6).

Similarly, the BAPC model predicts a decline in ASMR to about 0.69 per 100,000 by 2046 (Figure 5B; Supplementary Table S5). However, the absolute number of deaths is expected to rise sharply after 2040, culminating in over 80,000 deaths by 2046 (Figure 5D; Supplementary Table S6).

## 4 Discussion

This study analyzed temporal trends of asthma burden in China from 1990 to 2021 and projected incidence and mortality trends for the next 25 years. Using a combination of analytical methods, we explored spatiotemporal patterns and underlying age-period-cohort effects. The findings reveal a fluctuating decrease in incidence and a sustained decline in mortality. Age influenced incidence, while mortality increased with age. Gender differences were noted, with higher incidence in men, especially among older adults. Projections indicate continued declines in incidence and mortality by 2046, but an increase in total cases and deaths.

Asthma is one of the most common chronic diseases, affecting approximately 300 million individuals worldwide and causing an estimated 1,000 deaths per day (18). Over the past three decades, asthma severity and disease burden have varied across age groups, income levels, and regions (5, 19). China, with its substantial asthmatic population, ranks among the countries with the highest asthma mortality. As a rapidly industrializing nation with densely populated megacities, China faces evolving trends in asthma incidence and mortality driven by lifestyle shifts, environmental exposures, and demographic changes (6, 20, 21). According to China’s latest census, individuals aged over 60 accounted for 18,7% (264.02 million) of total population in 2020, a 2.2-fold increase from 8.57% in 1990, while those over 65 years comprised 13.5% (190.64 million) (21). These demographic changes underscore the urgent need for enhanced focus on chronic respiratory diseases like asthma to meet future survival and health improvement targets. Compared to the global average, the burden of asthma in China decreased significantly from 1990 to 2021. While the ASIR and ASMR were notably lower than global levels, the numbers of new cases and deaths ranked among the highest in the world (5, 22). Asthma incidence in China exhibited a general downward trend until 2004, then gradually increased over the next decade, likely due to enhanced disease detection following the severe acute respiratory syndrome (SARS) epidemic. Despite this, the overall ASIR decreased substantially over the past three decades, suggesting improvements in asthma management and possibly lifestyle changes that contributed to a reduction in the disease burden (23, 24).

Asthma is the leading chronic disease in children, responsible for one-third of pediatric emergency visits and the primary cause of preventable hospitalizations and school absenteeism (25). Children under the age of 10 consistently represented the primary age group for asthma incidence. A clear sex disparity exists: boys under the age of 13 have higher asthma rates, but adult women exhibit higher asthma rates than men globally (26). In China, children aligned with global patterns, but adult asthma prevalence differs (27), adult male incidence was higher, particularly among those aged 70–85. This disparity may be partially attributed to environmental pollution. A population-based study has shown that male patients are more susceptible to particulate matter (PM2.5, PM10), while older adults are more sensitive to carbon monoxide (CO) (20). Additionally, the higher incidence in adult men may be related to the elevated smoking rates among males in China. According to a report on health hazards of smoking in China (28), 26.6% of individuals aged over 15 years smoke, with 50.5% of men and only 2.1% of women smoking. Another survey conducted in China found that among individuals over 60, the smoking rate was 28.1%, with 52.9% of men and 3.1% of women smoking (29). Smoking is a significant risk factor for asthma, contributing to increased asthma exacerbations and poor asthma control (30–32). Moreover, as smoking is also a major risk factor for COPD, and given the genetic overlap between asthma and COPD (33), patients in the later stages of asthma are at increased risk for COPD, with many older adult patients exhibiting characteristics of both, severely impacting their quality of life (34). Given the high prevalence of smoking in Chinese males, targeted prevention and cessation efforts are essential to reducing asthma incidence in China.

Asthma mortality has steadily declined over the past 30 years, with a notable acceleration in this decline after 2004, likely attributable to improvements in medical interventions, management strategies (35, 36), and public awareness. Notably, mortality remained concentrated in older populations, while childhood asthma mortality has decreased, reflecting advancements in pediatric care. Older patients are more vulnerable to asthma-related mortality due to comorbidities such as COPD and cardiovascular disease, reducing lung function, and weakened immune systems (37, 38), increasing the risk of severe asthma exacerbations. Additionally, smoking history also increases the risk of fatal asthma attacks. Severe asthma, affecting approximately 5–10% of patients, is associated to higher mortality rates (39), and accounts for over 60% of total asthma healthcare costs (40, 41). Therefore, effective management of the older adult population and reasonable asthma treatment strategies are crucial.

The age-period-cohort analysis reveals the influence of age, period, and birth cohort on asthma incidence and mortality. The longitudinal age curve shows that age significantly impacts incidence at younger ages, while mortality rates continue to rise with age. The period ratio rate curve illustrates a decline in both incidence and mortality over time, suggesting the positive effects of advances in medical technology, disease management, and economic development on asthma in China. The cohort ratio curve reveals that earlier birth cohorts had a higher asthma risk, with more recent cohorts showing a lower risk. Similar patterns were observed for both men and women in this study.

Monitoring disease epidemics and predicting future patterns is crucial for effective disease prevention and control. According to the BAPC model, ASIR and ASMR of asthma are expected to drop to 330 per 100,000 and 0.69 per 100,000, respectively, by 2046. Nevertheless, the total number of new cases and deaths is expected to rise to over 4.5 million and 80,000, respectively, reflecting a more pessimistic outlook due to population aging and increased life expectancy, although it also contains the impacts of underlying co-morbidities, such as obesity, modern lifestyle and climate change (42). With a rising older adult population, China faces significant challenges in managing long-term population development. The growing older adult population necessitates strategic planning for health and social systems that support aging populations, particularly as population aging coincides with an increasing burden of noncommunicable diseases (43). Hence, it is necessary to promote community-based asthma screening, improve monitoring of air pollution, and strengthen health education in future policy development.

This study utilized multiple methodologies to process the most current data, enabling cross-temporal and spatial comparisons. The observed spatiotemporal trends and age-period-cohort effects provide valuable insights into asthma patterns in China. Several limitations should be acknowledged. First, while the GBD 2021 adjusted data for study heterogeneity and standardization, these revisions introduced some uncertainty in the analysis. Second, the COVID-19 pandemic may have influenced both data and projections, as a global decline in asthma cases during the pandemic lockdown and the wearing of masks outdoors (44), although the relationship between asthma and COVID-19 remains contentious (45). Third, demographic changes in China may affect current age-standardized rate analyses and introduce bias in future projections. Lastly, our study lacked geographic specificity due to the unavailability of provincial-level data.

## 5 Conclusion

This study highlights a decline in asthma incidence and mortality in China from 1990 to 2021, driven by improved management and healthcare. However, demographic shifts, particularly population aging poses ongoing challenges, with projections for 2046 indicate rising number of cases and deaths. Proactive measures focus on early detection, enhanced management, environmental interventions, and heightened disease awareness are critical to mitigating asthma’s public health impact.

## Data Availability

The original contributions presented in the study are included in the article/Supplementary material, further inquiries can be directed to the corresponding author.

## Footnotes

^1^ https://vizhub.healthdata.org/gbd-results/

## References

[1] Miller RL Grayson MH Strothman K. Advances in asthma: new understandings of asthma’s natural history, risk factors, underlying mechanisms, and clinical management. J Allergy Clin Immunol. (2021) 148:1430–1441. doi: 10.1016/j.jaci.2021.10.001, PMID: 34655640

[2] Mims JW. Asthma: definitions and pathophysiology. Int Forum Allergy Rhinol. (2015) 5:S2–6. doi: 10.1002/alr.21609, PMID: 26335832

[3] Wang S Liu H Yang P Wang Z Hu P Ye P. Exploring the genetic association of allergic diseases with cardiovascular diseases: a bidirectional Mendelian randomization study. Front Immunol. (2023) 14:1175890. doi: 10.3389/fimmu.2023.1175890, PMID: 37334359 PMC10272545

[4] Elias J. The relationship between asthma and COPD. Lessons Transgenic Mice Chest. (2004) 126:111S–6S. doi: 10.1378/chest.126.2_suppl_1.111S, PMID: 15302771

[5] Wang Z Li Y Gao Y Fu Y Lin J Lei X. Global, regional, and national burden of asthma and its attributable risk factors from 1990 to 2019: a systematic analysis for the Global Burden of Disease Study 2019. Respir Res. (2023) 24:169. doi: 10.1186/s12931-023-02475-6, PMID: 37353829 PMC10288698

[6] Yu J Xu L Han A Xie M. The epidemiology of asthma in mainland China: a systematic review and meta-analysis. BMC Public Health. (2024) 24:2888. doi: 10.1186/s12889-024-20330-1, PMID: 39434052 PMC11492516

[7] Huang K Yang T Xu J Yang L Zhao J Zhang X. Prevalence, risk factors, and management of asthma in China: a national cross-sectional study. Lancet. (2019) 394:407–418. doi: 10.1016/S0140-6736(19)31147-X, PMID: 31230828

[8] GBD Chronic Respiratory Disease Collaborators. Prevalence and attributable health burden of chronic respiratory diseases, 1990-2017: a systematic analysis for the Global Burden of Disease Study 2017. Lancet Respir Med. (2020) 8:585–596. doi: 10.1016/S2213-2600(20)30105-3, PMID: 32526187 PMC7284317

[9] Caffrey Osvald E Bower H Lundholm C Larsson H Brew BK Almqvist C. Asthma and all-cause mortality in children and young adults: a population-based study. Thorax. (2020) 75:1040–1046. doi: 10.1136/thoraxjnl-2020-214655, PMID: 32963117 PMC7677462

[10] Engelkes M de Ridder MA Svensson E Berencsi K Prieto-Alhambra D Lapi F. Multinational cohort study of mortality in patients with asthma and severe asthma. Respir Med. (2020) 165:105919. doi: 10.1016/j.rmed.2020.105919, PMID: 32174450

[11] Kuo CJ Chen VC Lee WC Chen WJ Ferri CP Stewart R. Asthma and suicide mortality in young people: a 12-year follow-up study. Am J Psychiatry. (2010) 167:1092–1099. doi: 10.1176/appi.ajp.2010.09101455 20634368

[12] Marois G Gietel-Basten S Lutz W. China’s low fertility may not hinder future prosperity. Proc Natl Acad Sci USA. (2021) 118:e2108900118. doi: 10.1073/pnas.2108900118, PMID: 34580226 PMC8501780

[13] Fang EF Xie C Schenkel JA Wu C Long Q Cui H. A research agenda for ageing in China in the 21st century (2nd edition): focusing on basic and translational research, long-term care, policy and social networks. Ageing Res Rev. (2020) 64:101174. doi: 10.1016/j.arr.2020.101174, PMID: 32971255 PMC7505078

[14] GBD 2021 Diseases and Injuries Collaborators. Global incidence, prevalence, years lived with disability (YLDs), disability-adjusted life-years (DALYs), and healthy life expectancy (HALE) for 371 diseases and injuries in 204 countries and territories and 811 subnational locations, 1990-2021: a systematic analysis for the Global Burden of Disease Study 2021. Lancet. (2024) 403:2133–2161. doi: 10.1016/S0140-6736(24)00757-8, PMID: 38642570 PMC11122111

[15] GBD 2021 Causes of Death Collaborators. Global burden of 288 causes of death and life expectancy decomposition in 204 countries and territories and 811 subnational locations, 1990-2021: a systematic analysis for the Global Burden of Disease Study 2021. Lancet. (2024) 403:2100–2132. doi: 10.1016/S0140-6736(24)00367-2, PMID: 38582094 PMC11126520

[16] Kim HJ Fay MP Feuer EJ Midthune DN. Permutation tests for joinpoint regression with applications to cancer rates. Stat Med. (2000) 19:335–351. doi: 10.1002/(sici)1097-0258(20000215)19:3<335::aid-sim336>3.0.co;2-z, PMID: 10649300

[17] Rosenberg PS Check DP Anderson WF. A web tool for age-period-cohort analysis of cancer incidence and mortality rates. Cancer Epidemiol Biomarkers Prev. (2014) 23:2296–2302. doi: 10.1158/1055-9965.EPI-14-0300, PMID: 25146089 PMC4221491

[18] Global Initiative for Asthma. (2024) Global strategy for asthma management and prevention (2024 update). Available online at: https://www.ginasthma.org (Accessed November 5, 2024)

[19] Shin YH Hwang J Kwon R Lee SW Kim MS. Global, regional, and national burden of allergic disorders and their risk factors in 204 countries and territories, from 1990 to 2019: a systematic analysis for the Global Burden of Disease Study 2019. Allergy. (2023) 78:2232–2254. doi: 10.1111/all.15807, PMID: 37431853 PMC10529296

[20] Shen W Ming Y Zhu T Luo L. The association between air pollutants and hospitalizations for asthma: an evidence from Chengdu. China Ann Transl Med. (2023) 11:65. doi: 10.21037/atm-22-6265, PMID: 36819554 PMC9929847

[21] National Bureau of Statistics of China. (2020). The seventh population census of China 2020. https://www.stats.gov.cn/sj/pcsj/rkpc/7rp/zk/indexch.htm (Accessed November 5, 2024)

[22] GBD 2019 Chronic Respiratory Diseases Collaborators. Global burden of chronic respiratory diseases and risk factors, 1990-2019: an update from the Global Burden of Disease Study 2019. EClinicalMedicine. (2023) 59:101936. doi: 10.1016/j.eclinm.2023.101936, PMID: 37229504 PMC7614570

[23] Chen ZH Wang PL Shen HH. Asthma research in China: a five-year review. Respirology. (2013) 18:10–19. doi: 10.1111/resp.12196, PMID: 24188199

[24] Zhou X Hong J. Pediatric asthma Management in China: current and future challenges. Paediatr Drugs. (2018) 20:105–110. doi: 10.1007/s40272-017-0276-7, PMID: 29222627

[25] Luo G Nkoy FL Stone BL Schmick D Johnson MD. A systematic review of predictive models for asthma development in children. BMC Med Inform Decis Mak. (2015) 15:99. doi: 10.1186/s12911-015-0224-9, PMID: 26615519 PMC4662818

[26] Chowdhury NU Guntur VP Newcomb DC Wechsler ME. Sex and gender in asthma. Eur Respir Rev. (2021) 30:210067. doi: 10.1183/16000617.0067-2021, PMID: 34789462 PMC8783601

[27] Liu M Gan H Lin Y Lin R Xue M Zhang T. Prevalence and disability-adjusted life year rates of asthma in China: findings from the GBD study 2019 of the G20. Int J Environ Res Public Health. (2022) 19:14663. doi: 10.3390/ijerph192214663, PMID: 36429381 PMC9690014

[28] Wang C Xiao D. The Writing Committee of 2020 Report on Health Hazards of Smoking in China. 2020 report on health hazards of smoking in China: an updated summary. Chinese Circulat J. (2021) 2021:937–952. doi: 10.3969/j.issn.1000-3614.2021.10.001

[29] Qingqing Z Shu C Jing F Ning W Wenjing W Jing W. Prevalence of smoking in adults aged 40 years and above in China, 2019-2020. Chin J Epidemiol. (2023) 44:735–42. doi: 10.3760/cma.j.cn112338-20230119-00035 37221061

[30] Li X Zhang Y Zhang R Chen F Shao L Zhang L. Association between E-cigarettes and asthma in adolescents: a systematic review and Meta-analysis. Am J Prev Med. (2022) 62:953–960. doi: 10.1016/j.amepre.2022.01.015, PMID: 35337694

[31] Silvestri M Franchi S Pistorio A Petecchia L Rusconi F. Smoke exposure, wheezing, and asthma development: a systematic review and meta-analysis in unselected birth cohorts. Pediatr Pulmonol. (2015) 50:353–362. doi: 10.1002/ppul.23037, PMID: 24648197

[32] Agache I Ricci-Cabello I Canelo-Aybar C Annesi-Maesano I Cecchi L Biagioni B. The impact of exposure to tobacco smoke and e-cigarettes on asthma-related outcomes: systematic review informing the EAACI guidelines on environmental science for allergic diseases and asthma. Allergy. (2024) 79:2346–2365. doi: 10.1111/all.16151, PMID: 38783343

[33] John C Guyatt AL Shrine N Packer R Olafsdottir TA Liu J. Genetic associations and architecture of asthma-COPD overlap. Chest. (2022) 161:1155–1166. doi: 10.1016/j.chest.2021.12.674, PMID: 35104449 PMC9131047

[34] Holtjer JCS Bloemsma LD Beijers RJHCG Cornelissen MEB Hilvering B Houweling L. Identifying risk factors for COPD and adult-onset asthma: an umbrella review. Eur Respir Rev. (2023) 32:230009. doi: 10.1183/16000617.0009-2023, PMID: 37137510 PMC10155046

[35] Wu X Gao S Lian Y. Effects of continuous aerobic exercise on lung function and quality of life with asthma: a systematic review and meta-analysis. J Thorac Dis. (2020) 12:4781–4795. doi: 10.21037/jtd-19-2813, PMID: 33145051 PMC7578506

[36] Xing S Feng S Zeng D. Effect of exercise intervention on lung function in asthmatic adults: a network meta-analysis. Ann Med. (2023) 55:2237031. doi: 10.1080/07853890.2023.2237031, PMID: 37563090 PMC10416742

[37] Rogliani P Laitano R Ora J Beasley R Calzetta L. Strength of association between comorbidities and asthma: a meta-analysis. Eur Respir Rev. (2023) 32:220202. doi: 10.1183/16000617.0202-2022, PMID: 36889783 PMC10032614

[38] Scelo G Torres-Duque CA Maspero J Tran TN Murray R Martin N. Analysis of comorbidities and multimorbidity in adult patients in the international severe asthma registry. Ann Allergy Asthma Immunol. (2024) 132:42–53. doi: 10.1016/j.anai.2023.08.021, PMID: 37640263

[39] Charles D Shanley J Temple SN Rattu A Khaleva E Roberts G. Real-world efficacy of treatment with benralizumab, dupilumab, mepolizumab and reslizumab for severe asthma: a systematic review and meta-analysis. Clin Exp Allergy. (2022) 52:616–627. doi: 10.1111/cea.14112, PMID: 35174566 PMC9311192

[40] Israel E Reddel HK. Severe and difficult-to-treat asthma in adults. N Engl J Med. (2017) 377:965–976. doi: 10.1056/NEJMra1608969, PMID: 28877019

[41] Khaleva E Rattu A Brightling C Bush A Bossios A Bourdin A. Development of Core outcome measures sets for paediatric and adult severe asthma (COMSA). Eur Respir J. (2023) 61:2200606. doi: 10.1183/13993003.00606-2022, PMID: 36229046 PMC10069873

[42] Sun Q Yu D Fan J Yu C Guo Y Pei P. Healthy lifestyle and life expectancy at age 30 years in the Chinese population: an observational study. Lancet Public Health. (2022) 7:e994–e1004. doi: 10.1016/S2468-2667(22)00110-4, PMID: 35926549 PMC7615002

[43] Wang H Chen H. Aging in China: challenges and opportunities. China CDC Wkly. (2022) 4:601–602. doi: 10.46234/ccdcw2022.130, PMID: 35919296 PMC9339359

[44] Dounce-Cuevas CA Flores-Flores A Bazán MS Portales-Rivera V Morelos-Ulíbarri AA Bazán-Perkins B. Asthma and COVID-19: a controversial relationship. Virol J. (2023) 20:207. doi: 10.1186/s12985-023-02174-0, PMID: 37679779 PMC10485988

[45] Skevaki C Karsonova A Karaulov A Xie M Renz H. Asthma-associated risk for COVID-19 development. J Allergy Clin Immunol. (2020) 146:1295–1301. doi: 10.1016/j.jaci.2020.09.017, PMID: 33002516 PMC7834224

