# Supplementary material for: Temporal trends and future projections of incidence rate and mortality for asthma in China: an analysis of the Global Burden of Disease Study 2021
Source: Front Med (Lausanne). 2025 Jan 29;12:1529636. doi: 10.3389/fmed.2025.1529636 (PMC11813779; doi:10.3389/fmed.2025.1529636)
Supplement: Supplementary file 1 [file Supplementary_file_1.docx]

Supplementary Materials for

**Temporal trends and future projections of incidence rate and mortality for asthma in China: an analysis of the Global Burden of Disease Study 2021**

**Supplemental materials Content**

**Table S1. APC and AAPC of age-standardized rate of incidence and mortality on asthma, 1990-2021.**

**Table S2. APC and AAPC of number of incidence and death on asthma, 1990-2021.**

**Table S3. Age-period-cohort effect of asthma incidence in China by sex, 1992-2021.**

**Table S4. Age-period-cohort effect of asthma mortality in China by sex, 1992-2021.**

**Table S5. Projected age-standardized rate of incidence and death by BAPC, 2022-2046.**

**Table S6. Projected number of incidence and death by BAPC, 2022-2046.**

**Figure S1. Age-period-cohort effects of asthma incidence and mortality for male in China, 1990-2021.**

**Figure S2. Age-period-cohort effects of asthma incidence and mortality for female in China, 1990-2021.**

**Figure S3. Trends in asthma incidence and mortality for male observed and predicted from 1990 to 2046.**

**Figure S4. Trends in asthma incidence and mortality for female observed and predicted from 1990 to 2046**

**Table S1**

**APC and AAPC of age-standardized rate of incidence and mortality on asthma, 1990-2021.**

| **Sex** | **ASIR** | | | **ASMR** | | |
| --- | --- | --- | --- | --- | --- | --- |
|  | **Segment** | **APC** | **AAPC** | **Segment** | **APC** | **AAPC** |
| Both | 1990-1996 | -1.63(-1.88,-1.39) | -1.21(-1.38,-1.04) | 1990-1999 | -3.65(-3.99,-3.31) | -4.40(-4.61,-4.20) |
|  | 1996-2000 | -3.90(-4.61,-3.19) |  | 1999-2004 | -1.44(-2.21,-0.67) |  |
|  | 2000-2005 | -4.89(-5.32,-4.45) |  | 2004-2007 | -10.53(-11.78,-9.27) |  |
|  | 2005-2010 | 2.41(1.92,2.90) |  | 2007-2014 | -5.36(-5.58,-5.14) |  |
|  | 2010-2014 | 1.77(0.95,2.60) |  | 2014-2021 | -3.77(-4.07,-3.48) |  |
|  | 2014-2021 | -0.83(-1.06,-0.60) |  |  |  |  |
|  |  |  |  |  |  |  |
| Female | 1990-1996 | -1.81(-2.06,-1.57) | -1.36(-1.50,-1.22) | 1990-2004 | -3.07(-3.18,-2.96) | -4.61(-4.75,-4.47) |
|  | 1996-2000 | -4.23(-4.94,-3.51) |  | 2004-2007 | -10.8(-11.89,-9.7) |  |
|  | 2000-2005 | -5.59(-6.04,-5.14) |  | 2007-2014 | -6.69(-6.89,-6.49) |  |
|  | 2005-2013 | 2.23(2.02,2.45) |  | 2014-2021 | -2.8(-3.09,-2.52) |  |
|  | 2013-2021 | -0.37(-0.57,-0.18) |  |  |  |  |
|  |  |  |  |  |  |  |
| Male | 1990-1995 | -1.38(-1.68,-1.09) | -1.06(-1.24,-0.87) | 1990-1999 | -3.71(-4.28,-3.13) | -4.15(-4.44,-3.86) |
|  | 1995-1998 | -2.94(-4.26,-1.61) |  | 1999-2004 | -0.17(-1.39,1.06) |  |
|  | 1998-2005 | -4.21(-4.43,-3.99) |  | 2004-2007 | -9.73(-11.35,-8.08) |  |
|  | 2005-2014 | 2.25(2.09,2.41) |  | 2007-2021 | -4.6(-4.71,-4.49) |  |
|  | 2014-2019 | -1.37(-1.87,-0.88) |  |  |  |  |
|  | 2019-2021 | -0.01(-1.55,1.55) |  |  |  |  |

APC: annual percent change; AAPC: average annual percentage change; ASIR: age-standardized incidence rate; ASMR: age-standardized mortality rate.

**Table S2**

**APC and AAPC of number of incidence and death on asthma, 1990-2021.**

| **Sex** | **Incidence** | | | **Death** | | |
| --- | --- | --- | --- | --- | --- | --- |
|  | **Segment** | **APC** | **AAPC** | **Segment** | **APC** | **AAPC** |
| Both | 1990-1996 | -2.93(-3.31,-2.54) | -1.89(-2.03,-1.75) | 1990-1998 | -1.79(-2.13,-1.45) | -1.70(-1.87,-1.53) |
|  | 1996-2005 | -5.69(-5.91,-5.48) |  | 1998-2004 | 0.19(-0.3,0.68) |  |
|  | 2005-2017 | 1.02(0.87,1.16) |  | 2004-2007 | -7.9(-8.95,-6.84) |  |
|  | 2017-2021 | -0.17(-0.91,0.57) |  | 2007-2014 | -1.81(-2,-1.62) |  |
|  |  |  |  | 2014-2021 | -0.34(-0.6,-0.08) |  |
|  |  |  |  |  |  |  |
| Female | 1990-1995 | -2.78(-3.08,-2.48) | -2.09(-2.26,-1.93) | 1990-2004 | -0.97(-1.07,-0.86) | -1.87(-2.00,-1.74) |
|  | 1995-2000 | -5.28(-5.68,-4.87) |  | 2004-2007 | -8.22(-9.25,-7.17) |  |
|  | 2000-2005 | -6.45(-6.82,-6.08) |  | 2007-2013 | -3.57(-3.81,-3.33) |  |
|  | 2005-2010 | 0.89(0.52,1.27) |  | 2013-2021 | 0.34(0.12,0.57) |  |
|  | 2010-2014 | -0.01(-0.67,0.65) |  |  |  |  |
|  | 2014-2018 | 1.52(0.81,2.25) |  |  |  |  |
|  | 2018-2021 | -0.49(-1.19,0.21) |  |  |  |  |
|  |  |  |  |  |  |  |
| Male | 1990-1995 | -2.42(-2.86,-1.97) | -1.70(-1.82,-1.59) | 1990-1998 | -2.15(-2.68,-1.61) | -1.51(-1.75,-1.27) |
|  | 1995-2005 | -5.31(-5.47,-5.15) |  | 1998-2004 | 0.79(0.04,1.55) |  |
|  | 2005-2016 | 1.35(1.21,1.50) |  | 2004-2007 | -7(-8.45,-5.52) |  |
|  | 2016-2021 | -0.25(-0.7,0.20) |  | 2007-2021 | -0.9(-1,-0.8) |  |

APC: annual percent change; AAPC: average annual percentage change.

**Table S3**

**Age-period-cohort effect of asthma incidence in China by sex, 1992-2021.**

| **Sex** | **Age Effect** | | **Drifts** | **Period Effect** | | **Cohort Effect** | |
| --- | --- | --- | --- | --- | --- | --- | --- |
|  | **Age** | **Longitudinal Rate,%** | **Local Drifts, % per year** | **Period** | **Period Rate Ratio** | **Cohort** | **Cohort Rate Ratio** |
| Both | <5 | 3285.05(2964.06,3640.79) | -0.34(-0.51,-0.17) | 1992-1996 | 1.47(1.41,1.54) | 1902-1926 | 3.84(1.56,9.46) |
|  | 5-9 | 1405.66(1268.34,1557.85) | -0.42(-0.59,-0.25) | 1997-2021 | 1.25(1.2,1.3) | 1907-1911 | 3.58(2.37,5.4) |
|  | 10-14 | 696.71(628.64,772.14) | -1.07(-1.29,-0.84) | 2002-2006 | 1(1,1) | 1912-1926 | 3.26(2.56,4.16) |
|  | 15-19 | 434.76(392.53,481.53) | -1.4(-1.67,-1.13) | 2007-2011 | 0.92(0.88,0.96) | 1917-1921 | 3.14(2.66,3.72) |
|  | 20-24 | 288.41(260.8,318.94) | -1.23(-1.52,-0.94) | 2012-2016 | 0.85(0.81,0.89) | 1922-1926 | 3.18(2.79,3.62) |
|  | 25-29 | 208.1(188.67,229.53) | -1.33(-1.63,-1.02) | 2017-2021 | 0.82(0.78,0.87) | 1927-1931 | 3(2.68,3.36) |
|  | 30-34 | 163.82(148.81,180.36) | -1.76(-2.1,-1.42) |  |  | 1932-1936 | 2.62(2.36,2.91) |
|  | 35-39 | 146.25(134.33,159.23) | -2.19(-2.55,-1.83) |  |  | 1937-1942 | 2.21(2,2.44) |
|  | 40-44 | 121.08(111.22,131.81) | -2.51(-2.89,-2.13) |  |  | 1942-1946 | 1.82(1.65,2) |
|  | 45-49 | 93.1(85.1,101.85) | -2.88(-3.27,-2.49) |  |  | 1947-1951 | 1.45(1.32,1.59) |
|  | 50-54 | 94.41(86.32,103.26) | -3.35(-3.74,-2.96) |  |  | 1952-1956 | 1.18(1.08,1.28) |
|  | 55-59 | 113.35(103.85,123.72) | -3.74(-4.11,-3.36) |  |  | 1957-1961 | 1(1,1) |
|  | 60-64 | 147.15(134.9,160.51) | -3.88(-4.24,-3.53) |  |  | 1962-1966 | 0.87(0.79,0.95) |
|  | 65-69 | 187.24(170.16,206.03) | -3.73(-4.08,-3.39) |  |  | 1967-1971 | 0.77(0.71,0.85) |
|  | 70-74 | 188.27(170,208.51) | -3.15(-3.53,-2.77) |  |  | 1972-1976 | 0.69(0.63,0.76) |
|  | 75-79 | 169.68(151.7,189.79) | -2.24(-2.73,-1.75) |  |  | 1977-1981 | 0.62(0.56,0.68) |
|  | 80-84 | 157.24(138,179.17) | -1.45(-2.17,-0.72) |  |  | 1982-1986 | 0.58(0.52,0.64) |
|  | 85-89 | 150.76(126.64,179.47) | -1.02(-2.25,0.23) |  |  | 1987-1991 | 0.57(0.51,0.63) |
|  | 90-94 | 150.36(112.66,200.67) | -0.92(-3.56,1.78) |  |  | 1992-1996 | 0.55(0.5,0.61) |
|  | >95 | 155.9(83.7,290.36) | -1.03(-8.58,7.14) |  |  | 1997-2001 | 0.48(0.43,0.53) |
|  |  |  |  |  |  | 2002-2006 | 0.42(0.38,0.47) |
|  |  |  |  |  |  | 2007-2011 | 0.49(0.44,0.54) |
|  |  |  |  |  |  | 2012-2016 | 0.54(0.49,0.6) |
|  |  |  |  |  |  | 2017-2021 | 0.44(0.4,0.49) |
| Male | <5 | 3139.45(2843.95,3465.65) | -0.32(-0.48,-0.17) | 1992-1996 | 1.43(1.37,1.5) | 1902-1926 | 3.42(1,11.65) |
|  | 5-9 | 1401.14(1269.37,1546.58) | -0.38(-0.53,-0.23) | 1997-2021 | 1.22(1.18,1.27) | 1907-1911 | 3(1.85,4.87) |
|  | 10-14 | 644.14(583.42,711.17) | -0.95(-1.15,-0.74) | 2002-2006 | 1(1,1) | 1912-1926 | 2.66(2.06,3.43) |
|  | 15-19 | 390.94(354.18,431.5) | -1.18(-1.43,-0.94) | 2007-2011 | 0.94(0.91,0.98) | 1917-1921 | 2.63(2.23,3.1) |
|  | 20-24 | 258.7(234.68,285.18) | -0.93(-1.21,-0.66) | 2012-2016 | 0.9(0.85,0.94) | 1922-1926 | 2.81(2.48,3.18) |
|  | 25-29 | 179.64(163.27,197.66) | -0.89(-1.18,-0.59) | 2017-2021 | 0.86(0.81,0.92) | 1927-1931 | 2.74(2.47,3.05) |
|  | 30-34 | 137.34(124.95,150.96) | -1.16(-1.49,-0.83) |  |  | 1932-1936 | 2.44(2.21,2.69) |
|  | 35-39 | 124.09(114.01,135.06) | -1.58(-1.94,-1.22) |  |  | 1937-1942 | 2.11(1.92,2.32) |
|  | 40-44 | 108.91(100.2,118.38) | -2.03(-2.39,-1.66) |  |  | 1942-1946 | 1.79(1.64,1.96) |
|  | 45-49 | 93.1(85.5,101.37) | -2.59(-2.96,-2.22) |  |  | 1947-1951 | 1.46(1.33,1.59) |
|  | 50-54 | 99.23(91.2,107.97) | -3.2(-3.57,-2.84) |  |  | 1952-1956 | 1.18(1.09,1.28) |
|  | 55-59 | 118.54(109.16,128.72) | -3.57(-3.93,-3.22) |  |  | 1957-1961 | 1(1,1) |
|  | 60-64 | 160.8(148.31,174.34) | -3.6(-3.93,-3.27) |  |  | 1962-1966 | 0.87(0.8,0.95) |
|  | 65-69 | 214.65(196.36,234.64) | -3.33(-3.65,-3.01) |  |  | 1967-1971 | 0.8(0.74,0.88) |
|  | 70-74 | 229.21(208.56,251.9) | -2.65(-3.01,-2.3) |  |  | 1972-1976 | 0.75(0.69,0.82) |
|  | 75-79 | 227.04(204.86,251.63) | -1.64(-2.11,-1.16) |  |  | 1977-1981 | 0.7(0.63,0.77) |
| **Sex** | **Age Effect** | | **Drifts** | **Period Effect** | | **Cohort Effect** | |
|  | **Age** | **Longitudinal Rate,%** | **Local Drifts, % per year** | **Period** | **Period Rate Ratio** | **Cohort** | **Cohort Rate Ratio** |
|  | 80-84 | 214.11(189.47,241.95) | -0.8(-1.56,-0.04) |  |  | 1982-1986 | 0.67(0.6,0.73) |
|  | 85-89 | 188.94(157.69,226.37) | -0.5(-1.95,0.96) |  |  | 1987-1991 | 0.66(0.6,0.73) |
|  | 90-94 | 167.42(115.54,242.6) | -0.75(-4.29,2.92) |  |  | 1992-1996 | 0.64(0.58,0.71) |
|  | >95 | 151(51.76,440.5) | -1.4(-13.2,12) |  |  | 1997-2001 | 0.57(0.51,0.63) |
|  |  |  |  |  |  | 2002-2006 | 0.51(0.46,0.56) |
|  |  |  |  |  |  | 2007-2011 | 0.57(0.52,0.63) |
|  |  |  |  |  |  | 2012-2016 | 0.63(0.57,0.7) |
|  |  |  |  |  |  | 2017-2021 | 0.52(0.47,0.58) |
| Female | <5 | 3317.87(2956.86,3722.95) | -0.37(-0.58,-0.15) | 1992-1996 | 1.52(1.45,1.6) | 1902-1926 | 4.54(2,10.31) |
|  | 5-9 | 1330.75(1185.74,1493.49) | -0.51(-0.72,-0.3) | 1997-2021 | 1.28(1.23,1.34) | 1907-1911 | 4.3(2.88,6.41) |
|  | 10-14 | 740.19(659.9,830.25) | -1.27(-1.54,-1.01) | 2002-2006 | 1(1,1) | 1912-1926 | 4.06(3.15,5.22) |
|  | 15-19 | 479.62(428.13,537.31) | -1.71(-2.03,-1.4) | 2007-2011 | 0.89(0.85,0.94) | 1917-1921 | 3.9(3.24,4.69) |
|  | 20-24 | 319.48(285.76,357.18) | -1.57(-1.9,-1.24) | 2012-2016 | 0.81(0.77,0.85) | 1922-1926 | 3.74(3.23,4.35) |
|  | 25-29 | 239.8(215.27,267.12) | -1.75(-2.09,-1.41) | 2017-2021 | 0.78(0.74,0.83) | 1927-1931 | 3.38(2.97,3.85) |
|  | 30-34 | 193.54(174.24,214.98) | -2.31(-2.68,-1.94) |  |  | 1932-1936 | 2.86(2.54,3.23) |
|  | 35-39 | 170.87(155.9,187.28) | -2.76(-3.16,-2.37) |  |  | 1937-1942 | 2.32(2.06,2.6) |
|  | 40-44 | 134.18(122.28,147.24) | -2.97(-3.39,-2.56) |  |  | 1942-1946 | 1.84(1.64,2.06) |
|  | 45-49 | 92.94(83.95,102.89) | -3.16(-3.59,-2.72) |  |  | 1947-1951 | 1.44(1.29,1.61) |
|  | 50-54 | 89.42(80.73,99.05) | -3.49(-3.93,-3.04) |  |  | 1952-1956 | 1.17(1.06,1.3) |
|  | 55-59 | 108.16(97.9,119.51) | -3.91(-4.35,-3.47) |  |  | 1957-1961 | 1(1,1) |
|  | 60-64 | 133.22(120.47,147.31) | -4.21(-4.63,-3.8) |  |  | 1962-1966 | 0.86(0.78,0.95) |
|  | 65-69 | 159.96(143.22,178.66) | -4.24(-4.64,-3.84) |  |  | 1967-1971 | 0.75(0.68,0.82) |
|  | 70-74 | 149.87(133.03,168.83) | -3.82(-4.26,-3.38) |  |  | 1972-1976 | 0.64(0.57,0.7) |
|  | 75-79 | 121.82(106.76,139.01) | -3.02(-3.57,-2.48) |  |  | 1977-1981 | 0.55(0.49,0.61) |
|  | 80-84 | 115.24(99.19,133.88) | -2.17(-2.92,-1.41) |  |  | 1982-1986 | 0.51(0.45,0.57) |
|  | 85-89 | 122(101.41,146.78) | -1.49(-2.68,-0.28) |  |  | 1987-1991 | 0.49(0.44,0.55) |
|  | 90-94 | 128.29(97.48,168.84) | -1.1(-3.5,1.37) |  |  | 1992-1996 | 0.48(0.43,0.53) |
|  | >95 | 133.93(78.13,229.56) | -0.95(-7.55,6.12) |  |  | 1997-2001 | 0.4(0.36,0.45) |
|  |  |  |  |  |  | 2002-2006 | 0.35(0.31,0.39) |
|  |  |  |  |  |  | 2007-2011 | 0.41(0.36,0.47) |
|  |  |  |  |  |  | 2012-2016 | 0.46(0.41,0.52) |
|  |  |  |  |  |  | 2017-2021 | 0.37(0.33,0.42) |

**Table S4**

**Age-period-cohort effect of asthma mortality in China by sex, 1992-2021.**

| **Sex** | **Age Effect** | | **Drifts** | **Period Effect** | | **Cohort Effect** | |
| --- | --- | --- | --- | --- | --- | --- | --- |
|  | **Age** | **Longitudinal Rate,%** | **Local Drifts, % per year** | **Period** | **Period Rate Ratio** | **Cohort** | **Cohort Rate Ratio** |
| Both | <5 | 7.24(6.12,8.55) | -12.59(-14.05,-11.1) | 1992-1996 | 1.6(1.55,1.65) | 1902-1926 | 14.53(12.85,16.43) |
|  | 5-9 | 1.61(1.36,1.91) | -11.38(-12.42,-10.33) | 1997-2021 | 1.24(1.21,1.28) | 1907-1911 | 11.65(10.75,12.62) |
|  | 10-14 | 0.95(0.81,1.12) | -9.8(-10.63,-8.97) | 2002-2006 | 1(1,1) | 1912-1926 | 10.19(9.52,10.91) |
|  | 15-19 | 0.64(0.55,0.75) | -7.91(-8.63,-7.18) | 2007-2011 | 0.66(0.64,0.68) | 1917-1921 | 8.74(8.2,9.32) |
|  | 20-24 | 0.61(0.53,0.69) | -6.03(-6.67,-5.39) | 2012-2016 | 0.5(0.48,0.51) | 1922-1926 | 6.92(6.5,7.36) |
|  | 25-29 | 0.61(0.55,0.69) | -5.12(-5.66,-4.57) | 2017-2021 | 0.4(0.38,0.41) | 1927-1931 | 5.37(5.06,5.71) |
|  | 30-34 | 0.6(0.55,0.67) | -4.67(-5.15,-4.18) |  |  | 1932-1936 | 4.13(3.89,4.38) |
|  | 35-39 | 0.72(0.66,0.78) | -4.6(-5.03,-4.17) |  |  | 1937-1942 | 3.17(2.99,3.36) |
|  | 40-44 | 0.83(0.77,0.89) | -4.75(-5.12,-4.38) |  |  | 1942-1946 | 2.34(2.2,2.49) |
|  | 45-49 | 0.86(0.8,0.91) | -4.97(-5.28,-4.66) |  |  | 1947-1951 | 1.74(1.64,1.84) |
|  | 50-54 | 1.02(0.96,1.08) | -5.19(-5.46,-4.92) |  |  | 1952-1956 | 1.29(1.21,1.37) |
|  | 55-59 | 1.24(1.17,1.31) | -5.52(-5.76,-5.29) |  |  | 1957-1961 | 1(1,1) |
|  | 60-64 | 1.57(1.48,1.66) | -5.6(-5.8,-5.4) |  |  | 1962-1966 | 0.77(0.72,0.82) |
|  | 65-69 | 2.22(2.1,2.36) | -5.57(-5.74,-5.41) |  |  | 1967-1971 | 0.62(0.58,0.67) |
|  | 70-74 | 3.75(3.53,3.98) | -5.37(-5.52,-5.23) |  |  | 1972-1976 | 0.48(0.44,0.52) |
|  | 75-79 | 5.55(5.23,5.9) | -5.12(-5.25,-4.99) |  |  | 1977-1981 | 0.38(0.34,0.43) |
|  | 80-84 | 8.27(7.78,8.79) | -4.66(-4.79,-4.52) |  |  | 1982-1986 | 0.31(0.27,0.35) |
|  | 85-89 | 12.66(11.89,13.49) | -4.11(-4.29,-3.93) |  |  | 1987-1991 | 0.23(0.2,0.26) |
|  | 90-94 | 14.6(13.61,15.67) | -3.75(-4.07,-3.43) |  |  | 1992-1996 | 0.16(0.14,0.19) |
|  | >95 | 12.38(11.11,13.79) | -3.47(-4.41,-2.53) |  |  | 1997-2001 | 0.1(0.08,0.12) |
|  |  |  |  |  |  | 2002-2006 | 0.05(0.04,0.06) |
|  |  |  |  |  |  | 2007-2011 | 0.02(0.02,0.03) |
|  |  |  |  |  |  | 2012-2016 | 0.01(0.01,0.02) |
|  |  |  |  |  |  | 2017-2021 | 0.01(0,0.01) |
| Male | <5 | 5.97(4.89,7.3) | -12.17(-13.94,-10.36) | 1992-1996 | 1.57(1.51,1.63) | 1902-1926 | 11.06(9.3,13.15) |
|  | 5-9 | 1.59(1.3,1.94) | -10.91(-12.15,-9.66) | 1997-2021 | 1.2(1.16,1.24) | 1907-1911 | 9.07(8.19,10.03) |
|  | 10-14 | 0.91(0.75,1.1) | -9.31(-10.29,-8.31) | 2002-2006 | 1(1,1) | 1912-1926 | 8.41(7.73,9.14) |
|  | 15-19 | 0.57(0.47,0.68) | -7.46(-8.32,-6.59) | 2007-2011 | 0.69(0.67,0.71) | 1917-1921 | 7.56(7,8.16) |
|  | 20-24 | 0.64(0.55,0.74) | -5.66(-6.42,-4.9) | 2012-2016 | 0.54(0.52,0.56) | 1922-1926 | 6.07(5.64,6.54) |
|  | 25-29 | 0.67(0.59,0.76) | -4.85(-5.49,-4.2) | 2017-2021 | 0.42(0.41,0.44) | 1927-1931 | 4.79(4.46,5.15) |
|  | 30-34 | 0.62(0.55,0.7) | -4.29(-4.86,-3.71) |  |  | 1932-1936 | 3.74(3.48,4.01) |
|  | 35-39 | 0.72(0.65,0.79) | -4.15(-4.67,-3.63) |  |  | 1937-1942 | 2.92(2.73,3.13) |
|  | 40-44 | 0.81(0.75,0.89) | -4.25(-4.69,-3.79) |  |  | 1942-1946 | 2.2(2.05,2.36) |
|  | 45-49 | 0.91(0.84,0.99) | -4.48(-4.85,-4.1) |  |  | 1947-1951 | 1.67(1.56,1.79) |
|  | 50-54 | 1.21(1.13,1.3) | -4.73(-5.05,-4.41) |  |  | 1952-1956 | 1.26(1.17,1.35) |
|  | 55-59 | 1.53(1.43,1.64) | -5.12(-5.4,-4.84) |  |  | 1957-1961 | 1(1,1) |
|  | 60-64 | 1.99(1.86,2.12) | -5.22(-5.46,-4.99) |  |  | 1962-1966 | 0.79(0.73,0.86) |
|  | 65-69 | 3.01(2.81,3.23) | -5.22(-5.41,-5.02) |  |  | 1967-1971 | 0.66(0.61,0.73) |
|  | 70-74 | 5.03(4.69,5.4) | -5.03(-5.2,-4.86) |  |  | 1972-1976 | 0.52(0.46,0.58) |
|  | 75-79 | 7.51(7,8.06) | -4.8(-4.96,-4.64) |  |  | 1977-1981 | 0.42(0.37,0.48) |
| **Sex** | **Age Effect** | | **Drifts** | **Period Effect** | | **Cohort Effect** | |
|  | **Age** | **Longitudinal Rate,%** | **Local Drifts, % per year** | **Period** | **Period Rate Ratio** | **Cohort** | **Cohort Rate Ratio** |
|  | 80-84 | 11.29(10.5,12.14) | -4.27(-4.44,-4.09) |  |  | 1982-1986 | 0.35(0.3,0.4) |
|  | 85-89 | 21.76(20.19,23.46) | -3.56(-3.8,-3.31) |  |  | 1987-1991 | 0.26(0.22,0.31) |
|  | 90-94 | 29.13(26.7,31.78) | -3.09(-3.56,-2.61) |  |  | 1992-1996 | 0.18(0.15,0.22) |
|  | >95 | 15.16(12.4,18.53) | -2.12(-4.35,0.16) |  |  | 1997-2001 | 0.12(0.09,0.15) |
|  |  |  |  |  |  | 2002-2006 | 0.06(0.04,0.08) |
|  |  |  |  |  |  | 2007-2011 | 0.03(0.02,0.04) |
|  |  |  |  |  |  | 2012-2016 | 0.02(0.01,0.02) |
|  |  |  |  |  |  | 2017-2021 | 0.01(0,0.01) |
| Female | <5 | 9.23(7.74,11.02) | -13.12(-14.64,-11.57) | 1992-1996 | 1.64(1.59,1.69) | 1902-1926 | 19.74(17.62,22.12) |
|  | 5-9 | 1.63(1.36,1.96) | -12.02(-13.14,-10.89) | 1997-2021 | 1.29(1.26,1.33) | 1907-1911 | 16.06(14.81,17.41) |
|  | 10-14 | 1(0.85,1.19) | -10.51(-11.4,-9.61) | 2002-2006 | 1(1,1) | 1912-1926 | 13.36(12.44,14.34) |
|  | 15-19 | 0.75(0.64,0.87) | -8.54(-9.31,-7.77) | 2007-2011 | 0.62(0.61,0.64) | 1917-1921 | 10.96(10.24,11.73) |
|  | 20-24 | 0.57(0.5,0.66) | -6.55(-7.23,-5.86) | 2012-2016 | 0.44(0.43,0.46) | 1922-1926 | 8.45(7.9,9.03) |
|  | 25-29 | 0.55(0.48,0.61) | -5.5(-6.08,-4.92) | 2017-2021 | 0.37(0.35,0.38) | 1927-1931 | 6.38(5.98,6.82) |
|  | 30-34 | 0.58(0.52,0.64) | -5.13(-5.64,-4.61) |  |  | 1932-1936 | 4.77(4.47,5.1) |
|  | 35-39 | 0.72(0.67,0.78) | -5.13(-5.58,-4.68) |  |  | 1937-1942 | 3.55(3.33,3.79) |
|  | 40-44 | 0.84(0.78,0.9) | -5.35(-5.73,-4.97) |  |  | 1942-1946 | 2.54(2.38,2.71) |
|  | 45-49 | 0.79(0.74,0.85) | -5.57(-5.89,-5.25) |  |  | 1947-1951 | 1.83(1.72,1.95) |
|  | 50-54 | 0.81(0.76,0.86) | -5.78(-6.06,-5.49) |  |  | 1952-1956 | 1.34(1.25,1.42) |
|  | 55-59 | 0.94(0.88,1) | -6.06(-6.32,-5.81) |  |  | 1957-1961 | 1(1,1) |
|  | 60-64 | 1.15(1.08,1.22) | -6.13(-6.35,-5.92) |  |  | 1962-1966 | 0.74(0.69,0.8) |
|  | 65-69 | 1.48(1.39,1.58) | -6.11(-6.29,-5.93) |  |  | 1967-1971 | 0.58(0.53,0.62) |
|  | 70-74 | 2.57(2.41,2.74) | -5.93(-6.09,-5.78) |  |  | 1972-1976 | 0.43(0.39,0.47) |
|  | 75-79 | 3.85(3.61,4.11) | -5.66(-5.79,-5.52) |  |  | 1977-1981 | 0.34(0.3,0.38) |
|  | 80-84 | 5.83(5.46,6.23) | -5.23(-5.36,-5.1) |  |  | 1982-1986 | 0.27(0.23,0.31) |
|  | 85-89 | 7.55(7.05,8.08) | -4.76(-4.93,-4.6) |  |  | 1987-1991 | 0.2(0.17,0.23) |
|  | 90-94 | 8.68(8.07,9.34) | -4.34(-4.63,-4.06) |  |  | 1992-1996 | 0.13(0.11,0.16) |
|  | >95 | 9.6(8.74,10.55) | -4(-4.7,-3.29) |  |  | 1997-2001 | 0.07(0.06,0.09) |
|  |  |  |  |  |  | 2002-2006 | 0.03(0.03,0.04) |
|  |  |  |  |  |  | 2007-2011 | 0.02(0.01,0.02) |
|  |  |  |  |  |  | 2012-2016 | 0.01(0.01,0.01) |
|  |  |  |  |  |  | 2017-2021 | 0(0,0.01) |

**Table S5**

**Projected age-standardized rate of incidence and death by BAPC, 2022-2046.**

| **Year** | **Both** | | **Male** | | **Female** | |
| --- | --- | --- | --- | --- | --- | --- |
|  | **incidence** | **death** | **incidence** | **death** | **incidence** | **death** |
| 2022 | 374.62 | 1.45 | 415.15 | 1.93 | 329.36 | 1.13 |
| 2023 | 374.57 | 1.40 | 414.57 | 1.86 | 330.05 | 1.10 |
| 2024 | 374.13 | 1.36 | 413.59 | 1.79 | 330.35 | 1.07 |
| 2025 | 373.36 | 1.32 | 412.30 | 1.73 | 330.33 | 1.04 |
| 2026 | 372.33 | 1.28 | 410.75 | 1.67 | 330.04 | 1.01 |
| 2027 | 371.07 | 1.23 | 408.98 | 1.61 | 329.53 | 0.99 |
| 2028 | 369.59 | 1.20 | 407.02 | 1.55 | 328.78 | 0.96 |
| 2029 | 367.94 | 1.16 | 404.91 | 1.49 | 327.84 | 0.93 |
| 2030 | 366.15 | 1.12 | 402.69 | 1.44 | 326.73 | 0.91 |
| 2031 | 364.24 | 1.09 | 400.35 | 1.39 | 325.49 | 0.88 |
| 2032 | 362.22 | 1.05 | 397.93 | 1.33 | 324.14 | 0.86 |
| 2033 | 360.11 | 1.02 | 395.43 | 1.29 | 322.68 | 0.84 |
| 2034 | 357.93 | 0.99 | 392.88 | 1.24 | 321.12 | 0.82 |
| 2035 | 355.69 | 0.96 | 390.29 | 1.20 | 319.50 | 0.79 |
| 2036 | 353.41 | 0.93 | 387.67 | 1.16 | 317.83 | 0.77 |
| 2037 | 351.10 | 0.90 | 385.02 | 1.12 | 316.13 | 0.75 |
| 2038 | 348.76 | 0.87 | 382.36 | 1.08 | 314.39 | 0.73 |
| 2039 | 346.40 | 0.84 | 379.70 | 1.04 | 312.62 | 0.72 |
| 2040 | 344.04 | 0.82 | 377.04 | 1.00 | 310.83 | 0.70 |
| 2041 | 341.67 | 0.80 | 374.39 | 0.97 | 309.03 | 0.68 |
| 2042 | 339.32 | 0.77 | 371.75 | 0.94 | 307.24 | 0.66 |
| 2043 | 336.96 | 0.75 | 369.13 | 0.91 | 305.43 | 0.65 |
| 2044 | 334.61 | 0.73 | 366.53 | 0.88 | 303.60 | 0.63 |
| 2045 | 332.27 | 0.71 | 363.95 | 0.85 | 301.76 | 0.62 |
| 2046 | 329.93 | 0.69 | 361.38 | 0.82 | 299.89 | 0.60 |

BAPC: Bayesian age-period-cohort.

**Table S6**

**Projected number of incidence and death by BAPC, 2022-2046.**

| **Year** | **Both** | | **Male** | | **Female** | |
| --- | --- | --- | --- | --- | --- | --- |
|  | **incidence** | **death** | **incidence** | **death** | **incidence** | **death** |
| 2022 | 3818361 | 27279 | 2197278 | 15837 | 1621083 | 11442 |
| 2023 | 3808077 | 27399 | 2186529 | 15821 | 1621548 | 11578 |
| 2024 | 3797109 | 27636 | 2175247 | 15872 | 1621862 | 11764 |
| 2025 | 3783166 | 27926 | 2161884 | 15950 | 1621282 | 11976 |
| 2026 | 3764657 | 28227 | 2145349 | 16031 | 1619308 | 12197 |
| 2027 | 3739098 | 28522 | 2123954 | 16101 | 1615144 | 12422 |
| 2028 | 3721287 | 28894 | 2108013 | 16209 | 1613274 | 12685 |
| 2029 | 3707601 | 29411 | 2094696 | 16397 | 1612905 | 13013 |
| 2030 | 3697350 | 30048 | 2083370 | 16655 | 1613980 | 13394 |
| 2031 | 3690030 | 30788 | 2073573 | 16969 | 1616457 | 13819 |
| 2032 | 3684721 | 31616 | 2064668 | 17333 | 1620053 | 14283 |
| 2033 | 3686848 | 32618 | 2060203 | 17793 | 1626645 | 14825 |
| 2034 | 3695982 | 33865 | 2059747 | 18388 | 1636235 | 15477 |
| 2035 | 3712119 | 35344 | 2063116 | 19112 | 1649003 | 16232 |
| 2036 | 3735399 | 37041 | 2070200 | 19956 | 1665199 | 17084 |
| 2037 | 3765826 | 38940 | 2080923 | 20912 | 1684903 | 18027 |
| 2038 | 3806359 | 41160 | 2097060 | 22052 | 1709299 | 19107 |
| 2039 | 3856678 | 43832 | 2118288 | 23449 | 1738390 | 20382 |
| 2040 | 3917120 | 46989 | 2144558 | 25115 | 1772562 | 21875 |
| 2041 | 3987947 | 50666 | 2175731 | 27058 | 1812216 | 23608 |
| 2042 | 4069156 | 54892 | 2211613 | 29289 | 1857543 | 25603 |
| 2043 | 4162557 | 59865 | 2253329 | 31907 | 1909228 | 27958 |
| 2044 | 4268860 | 65881 | 2300929 | 35083 | 1967931 | 30798 |
| 2045 | 4388910 | 73186 | 2354599 | 38968 | 2034311 | 34218 |
| 2046 | 4523286 | 82073 | 2414301 | 43744 | 2108985 | 38328 |

BAPC: Bayesian age-period-cohort.


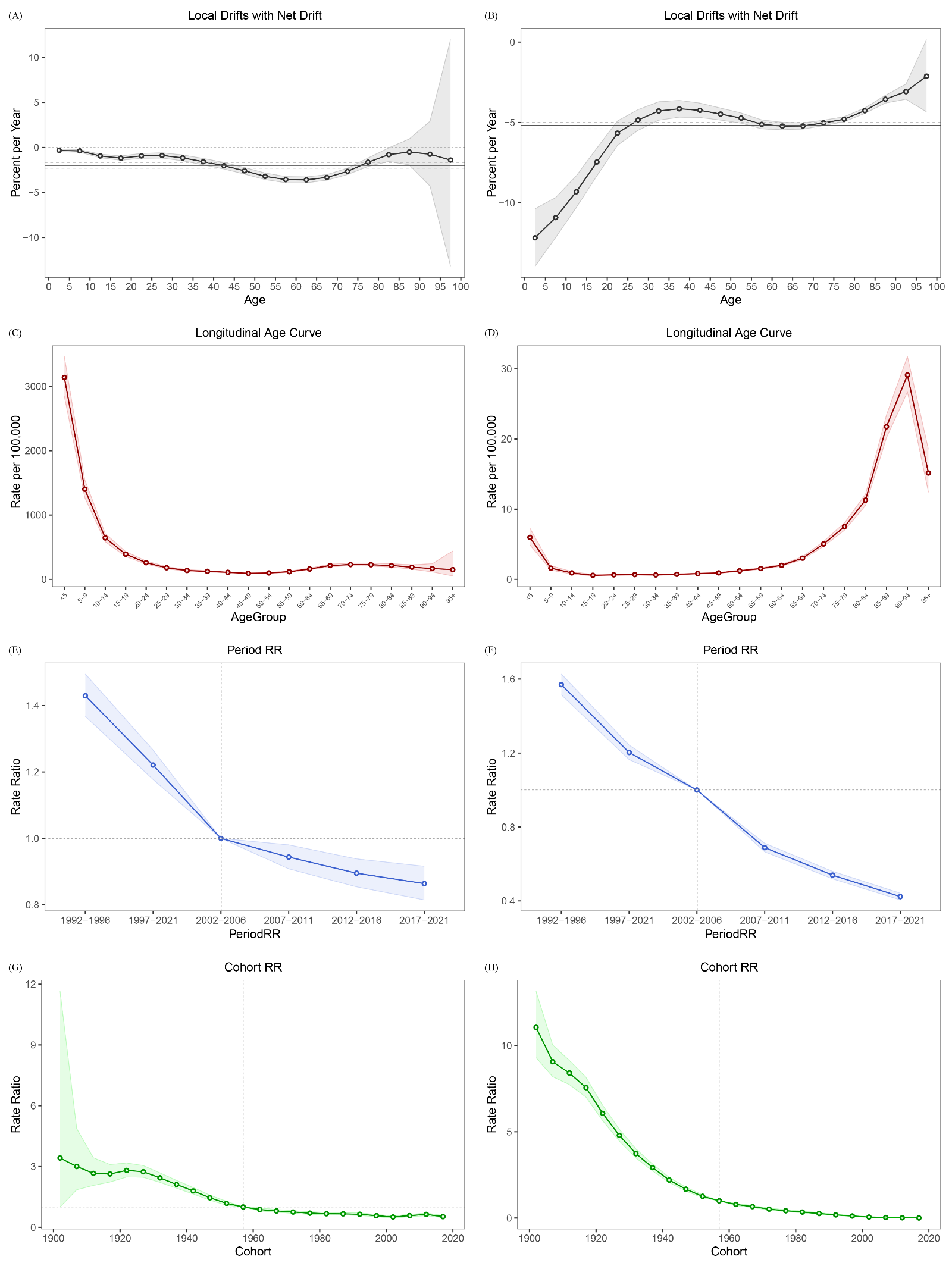


**Figure S1. Age-period-cohort effects of asthma incidence and mortality for male in China, 1990-2021.** (A) Local drifts and net drift of incidence; (B) Local drifts and net drift of mortality; (C) Longitudinal age curve of incidence; (D) Longitudinal age curve of mortality; (E) Period RR curve of incidence; (F) Period RR curve of mortality; (G) Cohort RR curve of incidence; (H) Cohort RR curve of mortality. RR: ratio rate.


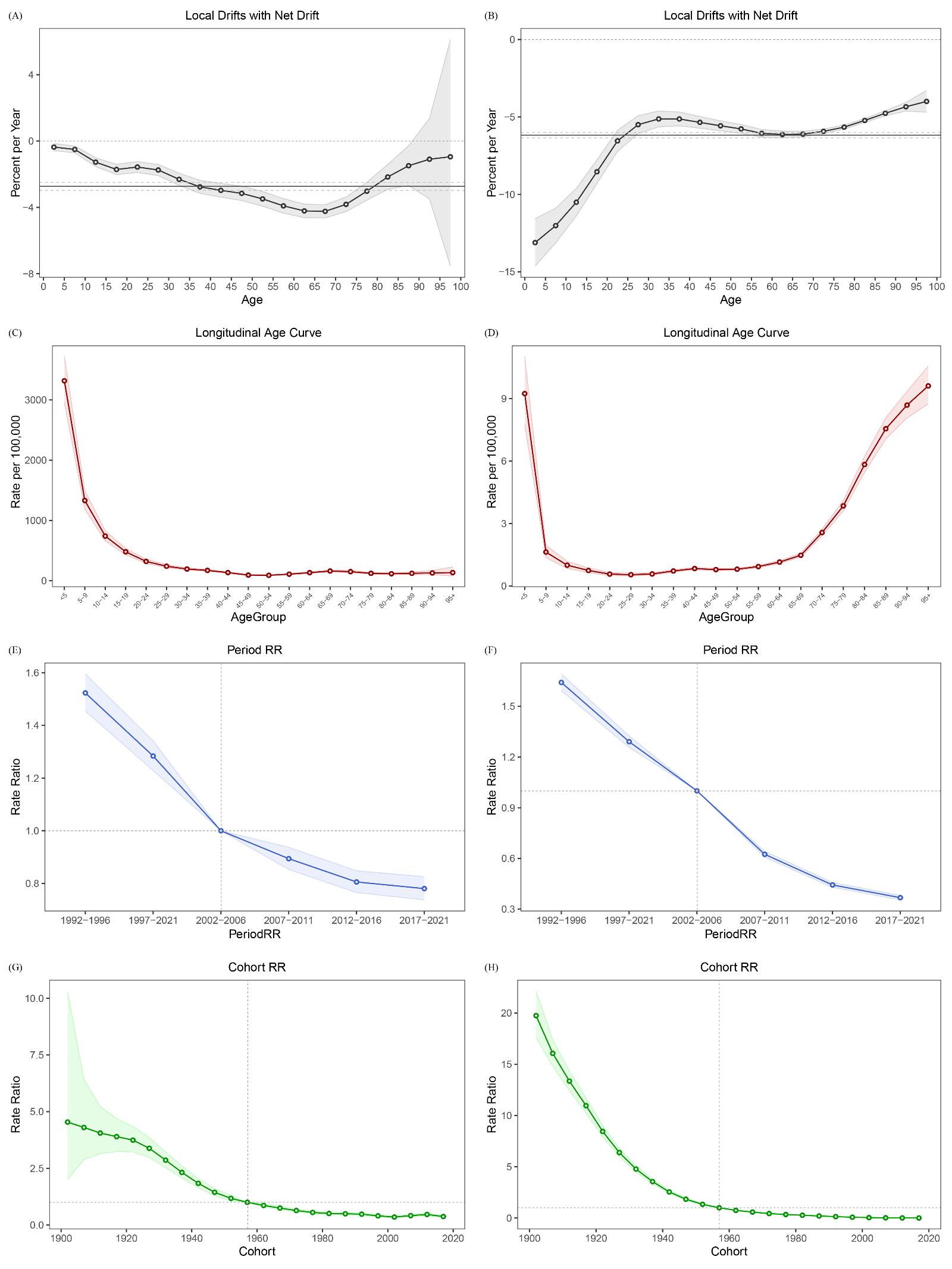


**Figure S2. Age-period-cohort effects of asthma incidence and mortality for female in China, 1990-2021.** (A) Local drifts and net drift of incidence; (B) Local drifts and net drift of mortality; (C) Longitudinal age curve of incidence; (D) Longitudinal age curve of mortality; (E) Period RR curve of incidence; (F) Period RR curve of mortality; (G) Cohort RR curve of incidence; (H) Cohort RR curve of mortality. RR: ratio rate.


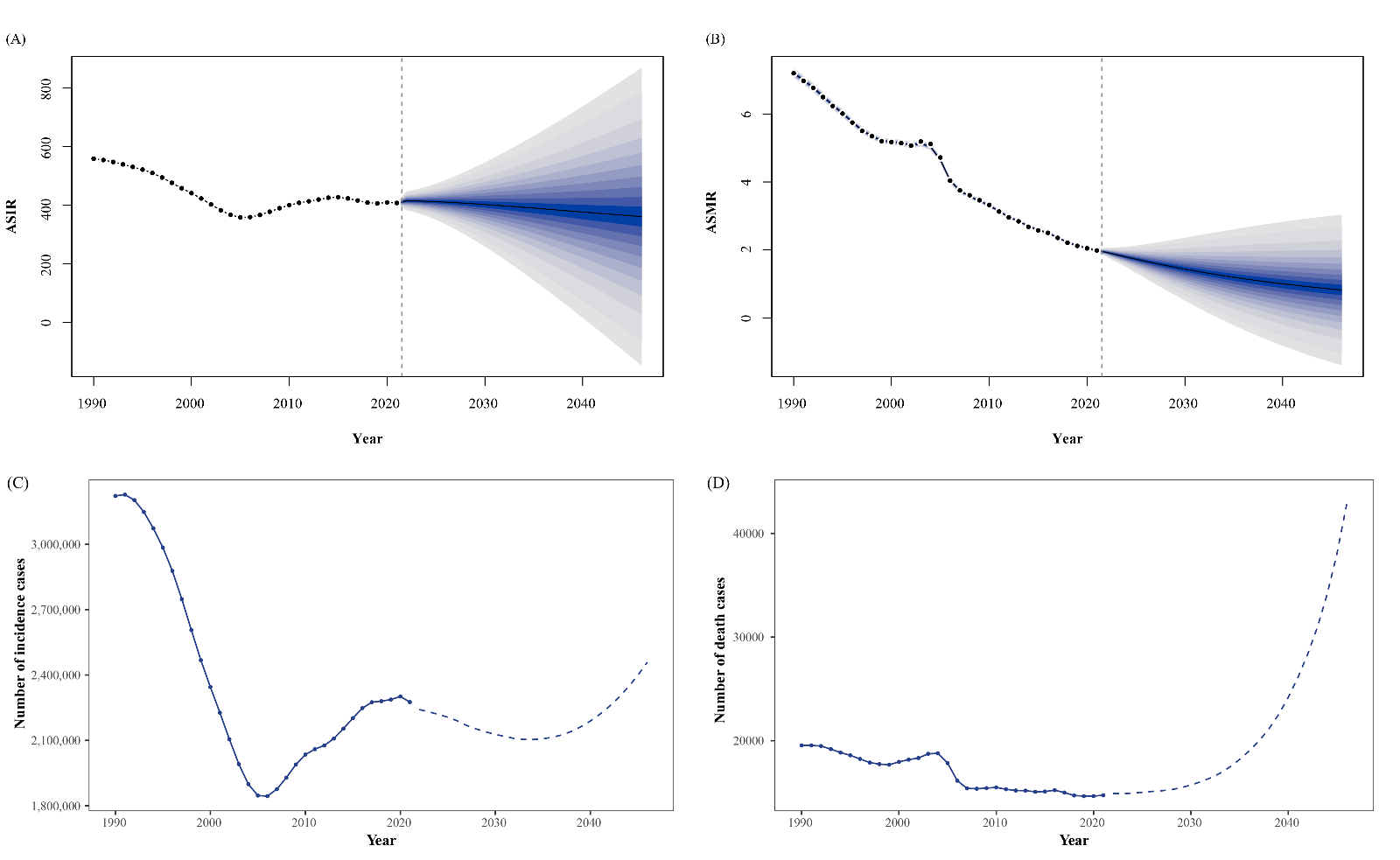


**Figure S3. Trends in asthma incidence and mortality for male observed and predicted from 1990 to 2046.** (A) Trends of age-standardized incidence rate (ASIR). (B) Trends of age-standardized mortality rate (ASMR). (C) Trends for the number of incidence cases. (D) Trends for the number of death cases.


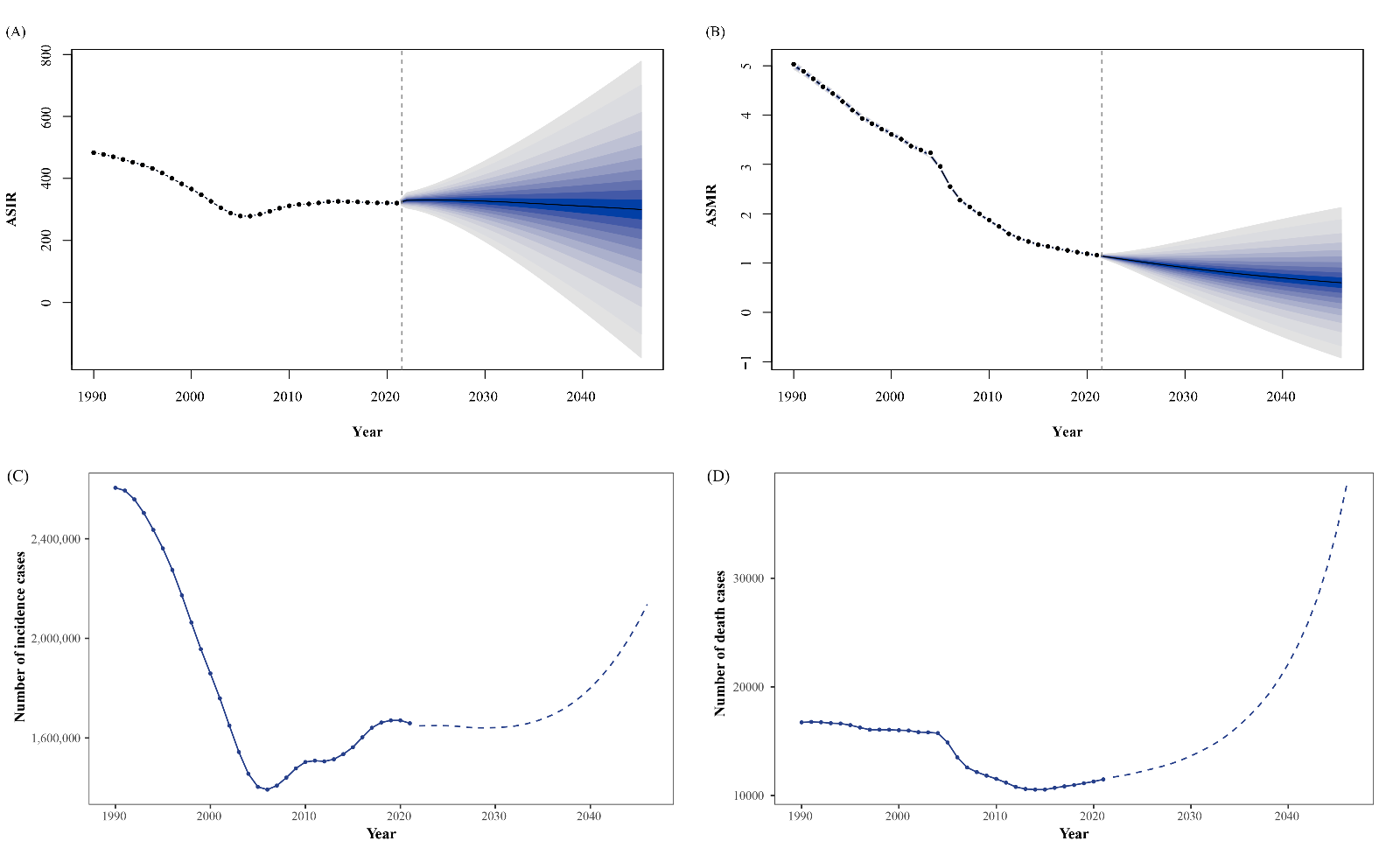


**Figure S4. Trends in asthma incidence and mortality for female observed and predicted from 1990 to 2046.** (A) Trends of age-standardized incidence rate (ASIR). (B) Trends of age-standardized mortality rate (ASMR). (C) Trends for the number of incidence cases. (D) Trends for the number of death cases.
